# Predictors of free-roaming domestic dogs' contact network centrality and their relevance for rabies control

**DOI:** 10.1038/s41598-021-92308-7

**Published:** 2021-06-18

**Authors:** Charlotte Warembourg, Guillaume Fournié, Mahamat Fayiz Abakar, Danilo Alvarez, Monica Berger-González, Terence Odoch, Ewaldus Wera, Grace Alobo, Elfrida Triasny Ludvina Carvallo, Valentin Dingamnayal Bal, Alexis Leonel López Hernandez, Enos Madaye, Filipe Maximiano Sousa, Abakar Naminou, Pablo Roquel, Sonja Hartnack, Jakob Zinsstag, Salome Dürr

**Affiliations:** 1 Veterinary Public Health Institute, Vetsuisse Faculty, University of Bern, Bern, Switzerland; 2 Royal Veterinary College, University of London, London, UK; 3 Institut de Recherche en Elevage pour le Développement, N’Djaména, Chad; 4 Universidad del Valle de Guatemala, Guatemala City, Guatemala; 5 Swiss Tropical and Public Health Institute, Basel, Switzerland; 6 College of Veterinary Medicine, Animal Resources and Biosecurity, Makerere University, Kampala, Uganda; 7 Kupang State Agricultural Polytechnic (Politeknik Pertanian Negeri Kupang), West Timor, Indonesia; 8 Animal Health Division, Agricultural Department of Sikka Regency, Flores, Indonesia; 9 Section of Epidemiology, Vetsuisse Faculty, University of Zurich, Zurich, Switzerland

**Keywords:** Diseases, Risk factors

## Abstract

Free roaming domestic dogs (FRDD) are the main vectors for rabies transmission to humans worldwide. To eradicate rabies from a dog population, current recommendations focus on random vaccination with at least 70% coverage. Studies suggest that targeting high-risk subpopulations could reduce the required vaccination coverage, and increase the likelihood of success of elimination campaigns. The centrality of a dog in a contact network can be used as a measure of its potential contribution to disease transmission. Our objectives were to investigate social networks of FRDD in eleven study sites in Chad, Guatemala, Indonesia and Uganda, and to identify characteristics of dogs, and their owners, associated with their centrality in the networks. In all study sites, networks had small-world properties and right-skewed degree distributions, suggesting that vaccinating highly connected dogs would be more effective than random vaccination. Dogs were more connected in rural than urban settings, and the likelihood of contacts was negatively correlated with the distance between dogs’ households. While heterogeneity in dog's connectedness was observed in all networks, factors predicting centrality and likelihood of contacts varied across networks and countries. We therefore hypothesize that the investigated dog and owner characteristics resulted in different contact patterns depending on the social, cultural and economic context. We suggest to invest into understanding of the sociocultural structures impacting dog ownership and thus driving dog ecology, a requirement to assess the potential of targeted vaccination in dog populations.

## Introduction

Free-roaming domestic dogs (FRDD) refer to domestic dogs (*canis familiaris*) that are owned by an individual or a community, and are allowed to roam without supervision all or part of the time. FRDD depend upon humans for food, and sometimes shelter and reproduction, and thus differ from ownerless dogs, or feral dogs, which do not depend upon humans anymore. In low and middle income countries, a large proportion of dogs are allowed to roam freely, and the majority of those are owned^1–5^. Ownerless dogs generally account for a small fraction of free-roaming dog populations, e.g. 8% in Bamako, Mali^6^, 3% in Tunisia^7^, 19% in Sri Lanka^8^, less than 1% in Iringa, Tanzania^9^ and less than 15% in N'Djaména, Chad^10,11^. Higher proportion of ownerless dogs were, however, observed in studies conducted in India (61.5%) and Bangladesh (40.5%)^12,13^. The capacity of owned FRDD to form stable groups of individuals that are frequently in contact with each other has been highly debated^14^. Recent studies show that dogs as social animals can form long-term relationships, and that association between dogs during foraging activities do not happen randomly^14,15^. However, it is also suggested that dogs are more likely to form stable packs when they are not socialized to humans and do not depend on them for food provision^16^. As such, there is a lack of knowledge about contact structures and social dynamics of FRDD that are owned by humans.

Dogs are known to be the main source of human rabies, a disease, which is annually responsible for an estimated 60,000 human deaths worldwide^17^. Rabies is a neurological disease resulting in an encephalitis which is almost always fatal after the onset of symptoms^18^. Transmission of rabies from dogs to humans and between dogs mostly occur through dog bites, but infection of open wounds, abrasions and mucous membranes with saliva can also lead to rabies transmission^18^. Rabies infection can develop into a furious or paralytic form. The furious form is characterized by hyperactivity, aggressiveness, and hyperaesthesia, while the paralytic form leads to a paralytic syndrome^18^. It has been repeatedly shown that mass vaccination of dogs can eliminate rabies from dog populations and therefore prevent human deaths^19–22^. The World Health Organization (WHO) currently recommends the random vaccination of at least 70% of dogs in susceptible dog populations. However, in its third Expert Consultation on Rabies, WHO stated that vaccination programs should be tailored to local characteristics of those populations^23^. Sustainable vaccination campaigns require considerable resources^24^ and refining vaccination recommendations, for example by targeting high-risk dog subpopulations to reduce the overall vaccination coverage needed to prevent outbreaks, would increase feasibility and effectiveness of those vaccination strategies^25,26^.

As rabies is transmitted by direct contact, the understanding of the potential impact of contact networks on the spread of rabies within dog populations could help tailoring vaccination strategies to the local context. Recent studies suggest that dog contact rates influence disease spread in FRDD populations^27^ and that highly connected dogs might play a critical role in rabies transmission^26,28^. Such important dogs in terms of rabies transmission could therefore be missed during vaccination campaigns. The age of a dog, the way of acquisition by its owner, and the ownership system (i.e. household versus community owned) were shown to influence the likelihood of a dog being vaccinated^29^. It was also suggested that targeting dogs with large individual home range could increase the effectiveness of vaccination programs^30–32^. Studies investigating FRDD ecology's impact on disease transmission are thus required^23,27^.

Combining network analysis and disease modelling, Wilson et al. in 2019 showed that it was possible to identify dogs presenting higher risk of disease transmission based on their centrality metrics^28^. Laager et al. in 2018 highlighted that targeting vaccination on a small number of the most central dogs in the contact network would reduce the vaccination coverage required for rabies elimination, and, therefore, increase its likelihood of success^26^. However, assessing the structure of a contact network in a dog population is labor-intensive, and would be inefficient to study in each dog population prior to vaccination campaigns. Instead, if characteristics associated with the dog's position in the network were identified, they could inform the design of risk-based vaccination strategies, allowing vaccinators to predict dogs with high centrality metrics for which vaccination should be prioritized^26,33^. The aim of this study was to compare the structure of dogs’ contact networks across different settings, and to identify dogs’ and dog owners’ characteristics associated with dogs’ network centrality. Using contact sensors, the study assessed contact patterns between owned FRDDs in eleven sites in four countries, namely Guatemala, Chad, Uganda and Indonesia, where canine rabies is endemic. Such information could be of high value to design more effective rabies vaccination campaigns.

## Results

### Study population

The study population includes a total of 714 owned dogs in 11 study sites in four countries. Data from 700 dogs were used for the analysis (Table 1). Dog characteristics of the study population have been described in another publication^34^. Briefly, the distribution of sex and age differed between countries, with a proportion of female dogs reaching 25%, 35%, 63%, 50%, in Chad, Guatemala, Indonesia and Uganda, respectively and a mean age of 1 and 2.5 years in Indonesia and Uganda (no reliable age data were available in Chad and Guatemala). Most of the dogs were kept for guarding purposes, some were also kept as hunting dogs, shepherd dogs, pets or for meat production (in Indonesia only)^34^. Nearly all owners were feeding their dogs daily^34^. Most dogs were allowed to roam freely at all time, but some dogs were restrained for the entire or part of the day and/or night. Some dogs that were always restrained but could be in contact with free-roaming dogs (i.e. chained outside) were included, as they were part of the contact network^34^. Information on the proportion of ownerless dogs was only available for Guatemala, where no ownerless dog in the study sites was estimated^35^. We do not expect a high proportion of ownerless dogs in the study sites of the three other countries.

**Table 1:** Network statistics of the 11 dog contact networks.

		Chad		Guatemala			Indonesia			Uganda		
		Rural 1	Rural 2	Rural 1	Rural 2	Urban/semi-urban	Rural 1	Rural 2	Urban/semi-urban	Rural 1	Rural 2	Urban/semi-urban
Number of dogs collared		21	25	61	125	117	52	65	100	17	8	124
Number of collars retrieved		21	25	57	123	113	52	65	99	15	8	122
Network size (% collars retrieved)		16 (76)	24 (96)	56 (98)	121 (98)	102 (90.3)	37 (71)	61 (94)	81 (82)	15 (100)	4 (50)	82 (67.2)
Size of the largest component (% network size)		16 (100)	24 (100)	56 (100)	116 (96)	93 (91)	35 (95)	59 (97)	81 (100)	11 (73)	2 (50)	77 (94)
Density		0.25	0.17	0.17	0.06	0.04	0.12	0.12	0.05	0.25	–	0.08
Average shortest path length^1^		2.5	2.6	2.1	3.2	3.8	3.2	2.5	4.0	1.8	–	3.0
Relative average shortest path length^1^		0.16	0.11	0.04	0.03	0.04	0.09	0.04	0.05	0.12	–	0.04
Clustering coefficient		0.56	0.45	0.39	0.38	0.27	0.5	0.46	0.42	0.58	–	0.43
Small world index		2.0	2.3	2.2	5.1	6.4	3.3	3.5	6.5	2.9	–	4.5
Degree	Median (IQR)	3.5 (2–4.25)	4 (2–5)	8 (6–13)	7 (4–10)	3 (2–5)	4 (2–6)	6 (4–9)	4 (2–5)	3 (2–5)	–	6 (3–10)
	CV	0.58	0.54	0.59	0.59	0.77	0.71	0.8	0.68	0.59	–	0.71
	Skewness coefficient	0.8	0.4	1.1	1.4	2.6	0.8	2.1	1.3	0.3		0.6
Relative degree	Median (IQR)	0.24 (0.13–0.29)	0.17 (0.09–0.22)	0.15 (0.11–0.24)	0.06 (0.04–0.08)	0.03 (0.02–0.05)	0.11 (0.06–0.17)	0.10 (0.07–0.15)	0.05 (0.02–0.06)	0.21 (0.14–0.36)	–	0.07 (0.04–0.12)
Betweenness	Median (IQR)	2.6 (0–16.5)	6.3 (0–23)	6.3 (1.6–35.6)	34.2 (4.3–117.8)	20.1 (0–123)	9.7 (0–33)	7.5 (0–31)	25.7 (0–127.5)	0.5 (0–3.1)	–	26.4 (0.8–86)
	CV	1.41	1.59	1.62	2.02	2.19	2.13	3.12	1.98	1.64	–	1.5
	Skewness coefficient	1.3	2.1	2.2	4.3	4.3	3.0	6.3	4.4	1.4	–	2.0
Normalized betweenness	Median (IQR)	0.02 (0–0.16)	0.02 (0–0.09)	0 (0–0.02)	0 (0–0.02)	0 (0–0.02)	0.02 (0–0.05)	0 (0–0.02)	0.01 (0–0.04)	0.01 (0–0.03)	–	0.01 (0–0.03)

The size of the network corresponds to the number of connected dogs in each study area (i.e. dogs with at least one contact). The relative degree correspond to the dog individual degree divided by the network size and the normalized betweenness correspond to the dog individual betweenness divided by the maximum betweenness in the network. CV = coefficient of variation, IQR = Interquartile range. ^1^Of the largest component. The characteristics of the dogs included in the current study were presented in Warembourg et al^34^.

### Contact network description

**Figure 1:**
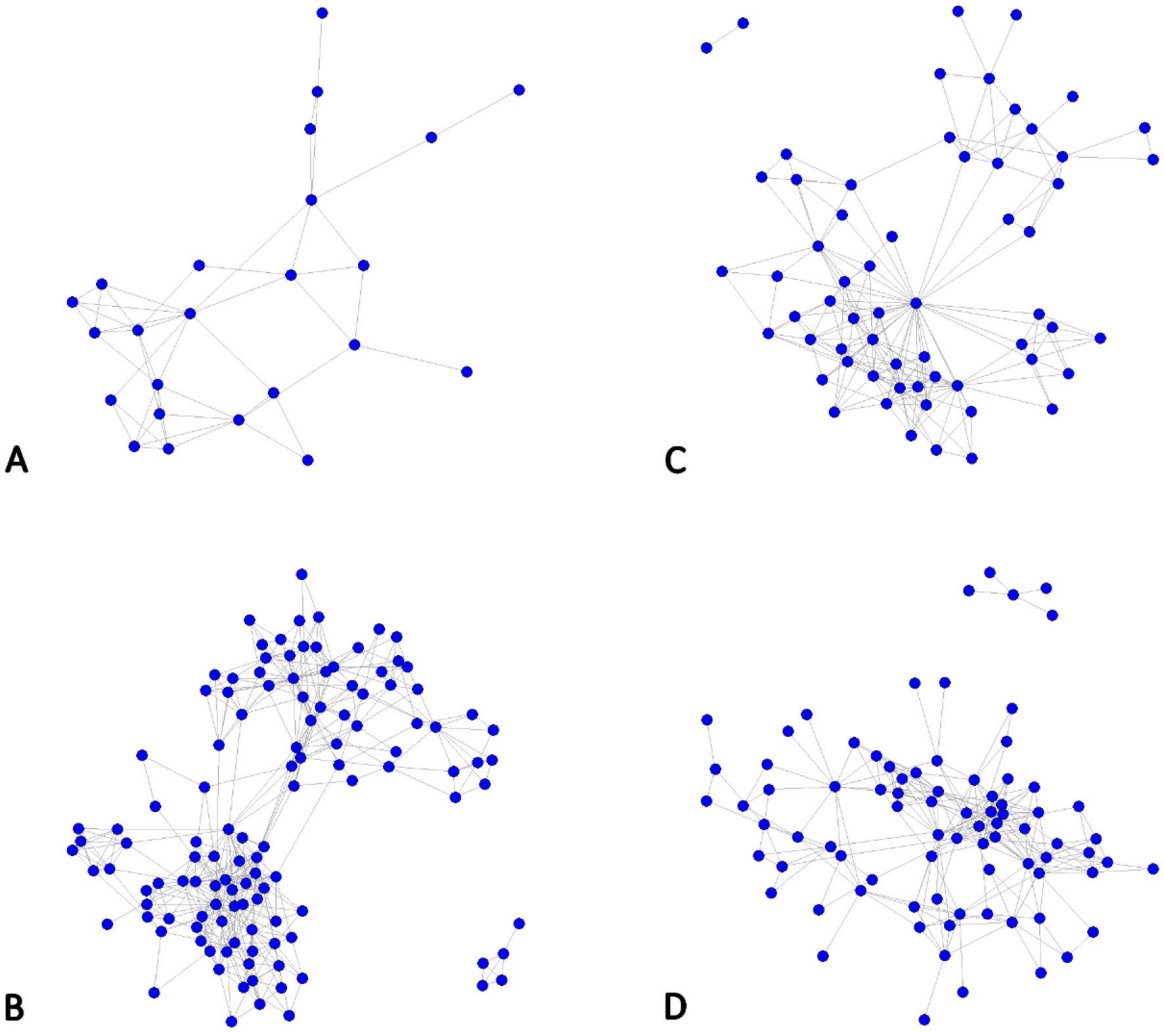
Undirected and unweighted dog contact networks. Nodes represent dogs and edges contacts between two dogs. Within these example networks it can be seen that some dogs are more connected than others. (**A**) Chad—rural 2 (network size = 24), (**B**) Guatemala—rural 2 (n = 121), (**C**) Indonesia—rural 2 (n = 61), (**D**) Uganda—urban/semi-urban (n = 82).

The structure of 11 undirected and unweighted networks was assessed, with dogs as nodes and contacts as edges between dogs (Fig. 1). A contact between two dogs was defined as the recording of at least one signal by at least one device over the study period. We chose to consider contacts received by at least one of the sensors because of the low sensitivity of some contact sensors. It was commonly observed that a sensor recorded many proximity events while the counterpart did not detect as many signals. The devices used in the study can capture signals up to a maximum of two to four meters, based on static tests (unpublished data).

Ten networks were further analyzed, since one Ugandan rural network was too small for further analysis (Supplementary Fig. S1). Network size, defined as the number of dogs with at least one connection to another dog (therefore excluding non-connected dogs), ranged from 15 to 123, representing 67% to 100% of the dogs for which contact sensor could be retrieved at the end of the data collection period (Table 1). It was not possible to differentiate truly isolated dogs (i.e. dogs not being in contact with any other dog during the study period) from those with malfunctioning contact sensors. In nine of the then networks, ≥ 90% of dogs (with the network size as denominator) were directly or indirectly connected within a single component, highlighting the high connectedness of the observed dog populations.

In order to compare degree (the number of individual dogs being in contact—Supplementary Table S1) or betweenness (number of time a given dog lies on the shortest path between two other dogs in the network) distributions across networks, relative degree (i.e. degree divided by the maximum possible degree (i.e. network size minus one)) and normalized betweenness (i.e. betweenness divided by the maximum possible betweenness in the network) were computed. The normalized betweenness corresponds to the fraction of all possible shortest paths in the network on which a given dog lies. These metrics were chosen because of their relevance for disease spread throughout the network. Dogs with a high degree are likely to infect a high number of dogs, and dogs with a high betweenness are likely to mediate rabies spread. Vaccinating such dogs could dramatically slow down disease transmission^36^.

**Figure 2:**
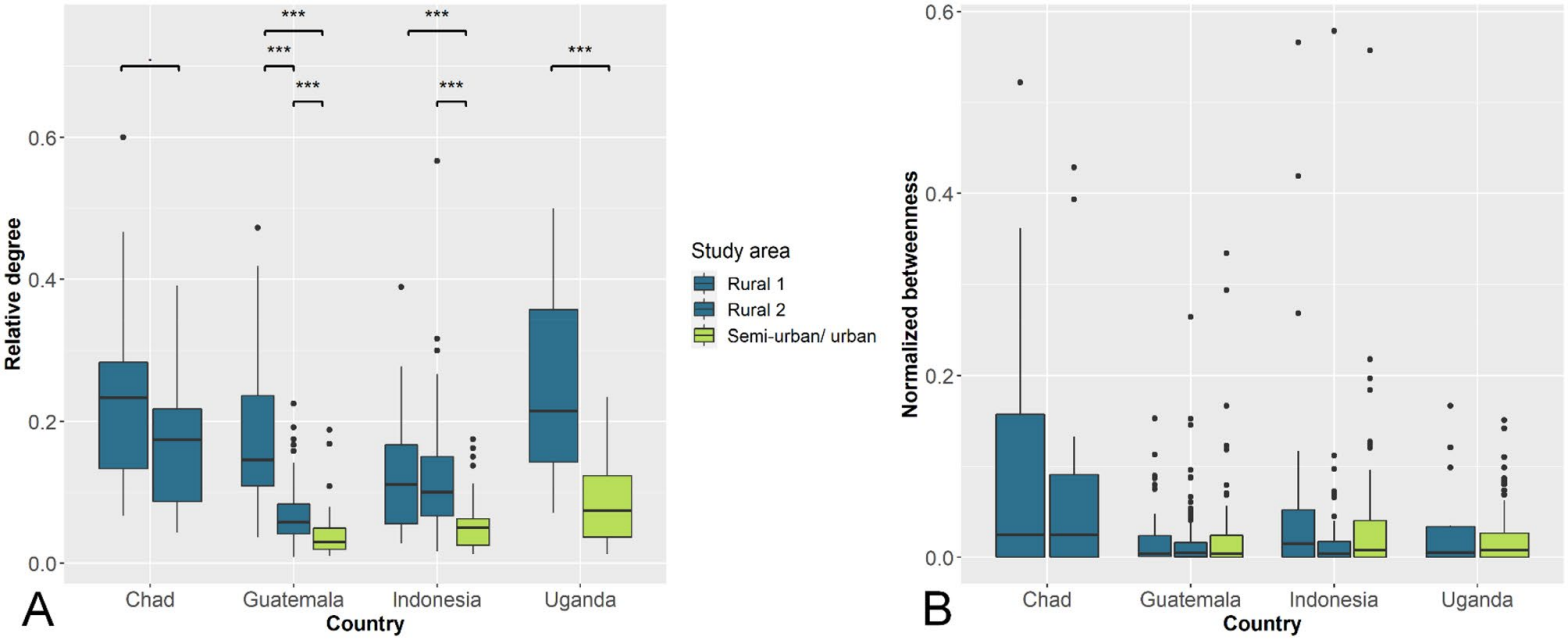
(**A**) Relative degree and (**B**) normalized betweenness distributions of ten dog contact networks in Chad, Guatemala, Indonesia and Uganda. Significance codes: 0.001 = ***, 0.01 = **, 0.05 = *, 0.1 = .

Within each country, relative degree and network density (i.e. the number of edges observed over the maximal possible number of edges) were lower in semi-urban and urban areas than in rural areas (Fig. 2A; Table 1). The distribution of the normalized betweenness was similar in all networks (Fig. 2B). Large heterogeneity of degree and betweenness amongst individual dogs was found in all networks. The degree and betweenness distributions were over-dispersed (high coefficients of variation ranging from 54 to 80% for the degree and 141% to 312% for the betweenness) and right-skewed (8 out 10 networks have skewness index above 0.5 for the degree, and all networks have a skewness index above 1 for the betweenness). The cumulative degree distributions of all networks display an exponential decay (Supplementary Fig. S2). These findings, together with a small-world index above one that was revealed in all investigated networks (Supplementary Table S2), are characteristics of small-world networks^37^. All networks were characterized by a high clustering coefficient (i.e. probability that adjacent nodes of a node are also connected) and short path lengths between nodes (i.e. number of edges on the shortest path between a pair of nodes). This has consequences for disease transmission since infection spreads faster in small-world networks compared to random networks^36,38–40^.

### Predictors for dog and household centrality in the network (node-level analysis)

To identify predictors of dogs’ centrality in a network, permutation-based linear regression models (PBLM) with degree or betweenness as the response variable were applied for each of the ten networks. Independent variables included sex, age, body condition score (BCS), and role of the dog (guardian, hunting, shepherd and source of meat) (Supplementary Table S3). To explore the potential impact of the owner's social network on the dog network, PBLMs were also used to explore whether owner related characteristics were associated with households’ centrality. For this, a household-level network was built, in which an edge was formed between two households if at least one dog from each of these households were in contact. The owners' characteristics investigated in the household network were wealth (for Chad, Uganda and Guatemala) or income (for Indonesia), education level, religion and ethnicity of the dog owners (Supplementary Table S4). Categorization of the wealth level is explained in Supplementary Methods, Supplementary Tables S5–S9, and Supplementary Fig. S3. All models were controlled for the length of time the dog is allowed roaming freely (FRT—dog-level PBLM only), the number of dogs collared in the same household (NDC—dog- and household-level PBLM), and the distance from the household to the centroid of the study area (Distance—dog- and household-level PBLM). This latter was included to correct for potential edge effect, in case contacts with non-collared dogs were missed for dogs living close to the edge of the study area.

**Figure 3:**
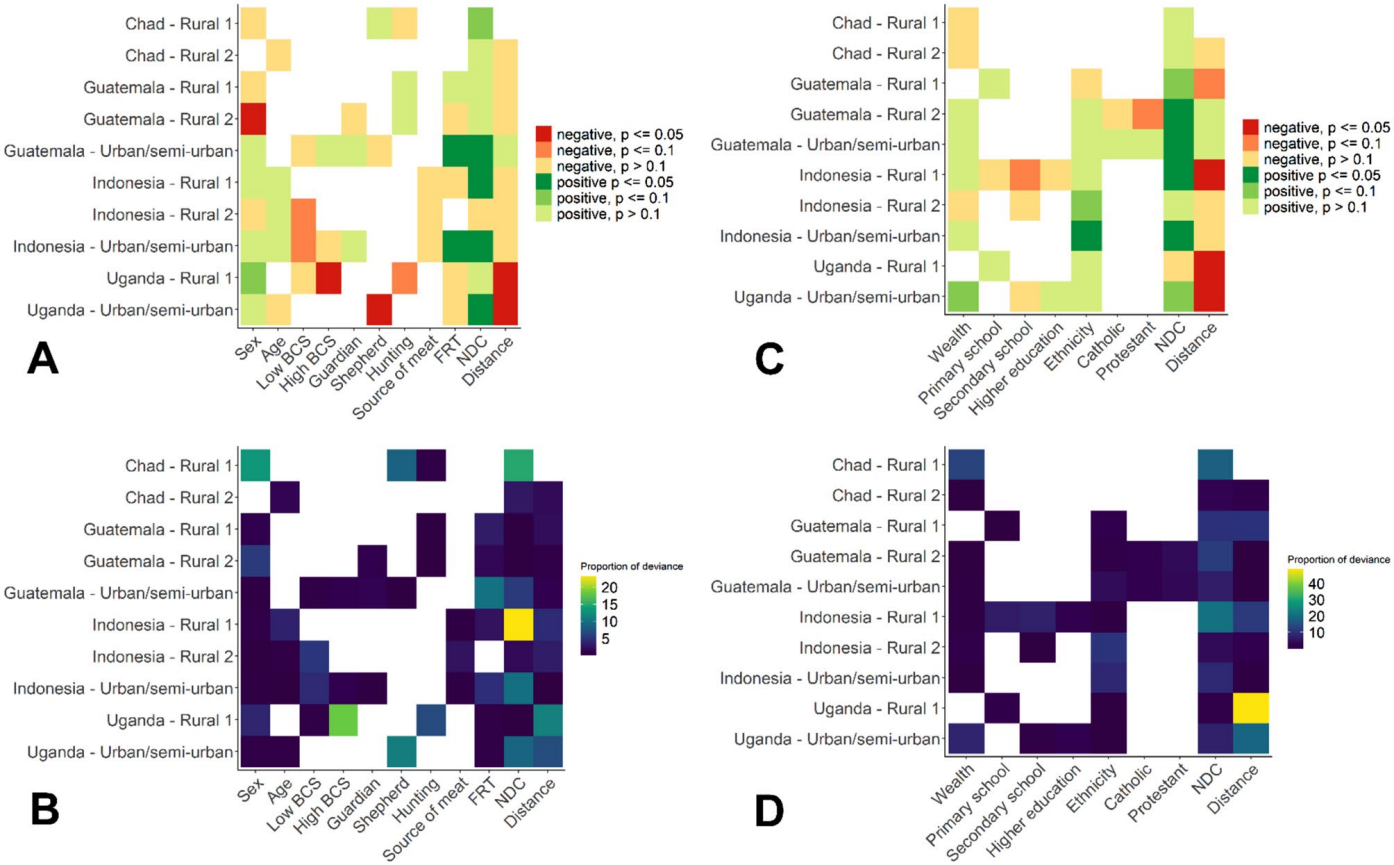
Permutation-based linear model (PBLM) results with degree as response variable. (**A**) Significance and direction of coefficients of the dog-level networks. (**B**) Proportion of deviance of the PBLM explained by each variable in the dog-level networks. (**C**) Significance and direction of coefficients of the household-level networks. (**D**) Proportion of deviance explained by each variable in the household-level networks. Empty field denote variables that were not explored or were not selected for the best models by the PBLM (see methods). Details on the values of the coefficients and *p* values are presented in the Supplementary Tables S10–S21. Dog-level factors (see also Supplementary Table S3) are: dog's sex (Sex—0: male (baseline), 1: female); body condition score (BCS) of 2 and lower (Low BCS), BCS of 4 and higher (High BCS) with the baseline of BCS = 3; being a guardian dog (Guardian, dummy variable), hunting dog (Hunting, dummy variable), shepherd dog (Shepherd, dummy variable) or raised for meat (Source of meat, dummy variable); free-roaming time (FRT—range from 0 to 10); number of dogs collared per household (NDC, contiguous variable); and distance per 100 m from dogs home to the centroid of the study site (Distance, continuous variable). Household-level factors (see also Supplementary Table S4) are: wealth category based on the Multiple Factor Analysis or the income when available (Wealth, with the lowest level (i.e. poorest) being the baseline); owner finalizing primary school (Primary school), finalizing secondary school (Secondary school), finalizing professional training or university (Higher Education), with absence of formal education being the baseline; owner belonging to the main local ethnicity (Ethnicity, dummy variable), being catholic (Catholic, dummy variable), being evangelic (Evangelic, dummy variable); number of dogs collared per household (NDC, contiguous variable); and distance per 100 m from household to the centroid of the study area (Distance, continuous variable).

Results from the dog- and household-level PBLM displayed high heterogeneity between study sites for degree as response variable (Fig. 3, refer to Supplementary Tables S10–S15 for details). The set of predictors selected for the best PBLM varies from one site to another and some predictors were found to be positively associated in one site, and negatively associated in another site. For example, female dogs were associated with lower degree than males in a rural site in Guatemala (rural 2), but higher degree in a rural site in Uganda (rural 1) (Fig. 3A). Dogs with ideal BCS tended to have higher degree than dogs with BCS under or above the ideal score, but not in all networks. Being a shepherd dog was negatively associated with degree in Uganda, whereas this variable did not have any impact on degree in two other networks. Other dog role categories and the dog's age did not significantly influence the dogs' degree in none of the networks. In the household networks, being from the main local ethnicity tended to be positively associated with higher degree of the households (in one network significantly), which may indicate that dogs living in households of the main ethnicity are more connected than others (Fig. 3C). A trend towards higher degree was detected for the household wealth (the higher the wealth, the more connected), but only in one network, whereas finalizing secondary school and being protestant tended to be negatively associated in one network each.

In contrast to this heterogeneity, the daily duration a dog was allowed to freely roam and the number of dogs collared in the same household were generally positively associated with the degree in most, although not all, networks (positive coefficient in 9 networks and p < 0.1 in 5 networks; for details consult Tables S5–S7). The distance from the centroid of the study area to the dog's home was negatively associated with the degree in most sites, with a significance found in both Ugandan sites. This justifies the inclusion of this control variable to adjust for the edge effect.

The proportion of deviance explained by the variables included in the best models ranged from 0.001 to 23.3% (median = 1.5%) for the dog-level networks and from 0.002 to 48.6% (median = 2.2%) for the household-level network. Mostly control variables (number of dogs collared or distance to the centroid) but also BCS and sex in single networks, substantially contributed in explaining the deviance of the models (Fig. 3B,D). The small proportion of the deviance of the model explained by the investigated variables suggests that non-measured factors might highly influence the network metrics.

The results of the PBLM with betweenness as response variable are similar to those of the degree and presented in the supplementary information (Supplementary Fig. S4 and Supplementary Tables S16–S21).

### Predictors for contacts between dogs and households (edge-level analysis)

To assess whether dog—or owner—related characteristics were associated with a contact between a given pair of dogs—or households—we applied multiple regression quadratic assignment procedure (MRQAP). The aim of the analysis was to assess whether dogs are more likely to be connected if they share similarities in respect to certain variables. Variables explored were sex, age, BCS, reason for keeping the dog (guardian, hunting, shepherd), free-roaming time period and distance between the households for the dog-level networks, and similarity in wealth categories (for Chad, Uganda, Guatemala) or income (for Indonesia), education level, religion, ethnicity and distance between the households for the household-level networks (Supplementary Tables S22 and S23).

**Figure 4:**
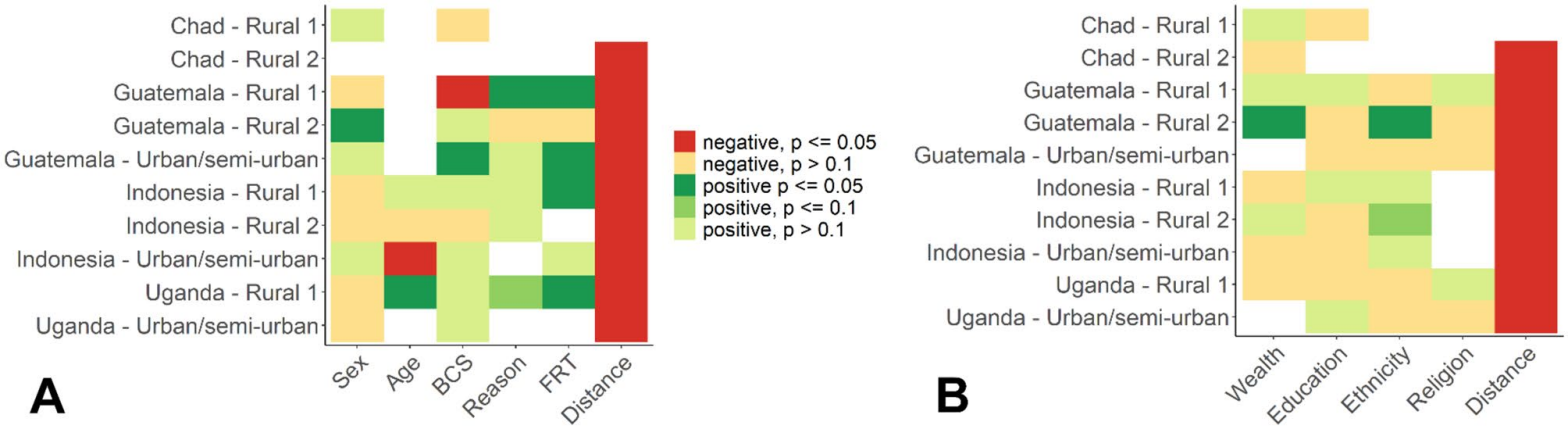
Odds ratios derived from the multiple regression quadratic assignment procedure (MRQAP) between dogs (**A**) or households (**B**) having the same level per variable investigated and being in contact. Positive association: OR > 1, negative association: OR < 1. Empty field denote variables that were not explored or were not selected for the best models by the MRQAP (see methods). Details on the values of the coefficients and *p* values are presented in the Supplementary Tables S24–S27. Dog-level factors (see also Supplementary Table S22) are: sex (male versus female), age category (more or less than two years old), BCS category (more or less than 2), reason for keeping the dog (guardian, hunting, shepherd, source of meat), free-roaming time (FRT, always free-roaming, free-roaming by day, by night, a few hours per day or never), and distance per 100 m between the households (Distance, continuous variable). Household-level factors (see also Supplementary Table S23) are: wealth category (Wealth, cluster 1 to 4), education level (Education: no formal education, primary education, secondary education, higher education), ethnicity (Ethnicity, various levels depending on the study site), religion (Religion, various levels depending on the study site) and distance per 100 m between the households (Distance, continuous variable).

The results, again, revealed differences in identifying predictors for contacts across networks, except for the distance between dog owners’ households which was always negatively associated with the occurrence of contacts in both the dog- and household-level networks (Fig. 4, for details consult Supplementary Tables S24–S27). This suggests that networks were spatially driven, with dogs being more likely to be in contact if their owners’ households were close, as also shown in earlier studies^41^. Likewise, dogs roaming freely during the same periods of the day or night were more likely to be in contact, in most of the explored networks. Dogs kept for the same purpose (guardian, hunting dog, shepherd dog or raised for meat) tended to be more likely be in contact with one another than dogs kept for a different purpose. In addition, some characteristics (sex, BCS and age) showed different influence across networks, being associated with a higher likelihood of contact in some networks and a lower likelihood or even not associated in other networks. For instance, dogs of the same sex were more likely to be in contact in Guatemala (rural 2), whereas sex has no impact in the rest of the networks.

At the household-level, dogs whose owners belonged to similar wealth categories or had the same ethnicity background were more likely to be in contact in one (Guatemala, rural 2) and two (Guatemala, rural 2 and Indonesia, rural 2) study sites, respectively, even after controlling for spatial locations of households. On the other hand, education and religion did not show much of an effect in any network.

## Discussion

Understanding the ecology of the host population is essential for effective disease control. For rabies, several studies call for improved knowledge on dog ecology to plan vaccination campaigns more effectively^5,27,29,30,32,42–44^. With this study, we made an important step into understanding the structure of FRDD contact networks, and assessing the factors shaping those networks. The large number of dogs involved in our contact networks (sometimes more than a hundred dogs) and the diversity of settings compared to other studies^26,32,33^, is a clear strength of this study. The ten dog contact networks investigated in displayed strong similarities in terms of structure, but were heterogeneous regarding factors shaping these structures and associated with dogs' centrality.

The contact networks showed a similar structure with almost all dogs connected directly or indirectly with one another. The differences in the size of the networks reflect the variability of dog density across study sites. The dog density was probably influenced by the human density, the local setting (i.e. urban, peri-urban or rural) as well as cultural factors. All networks showed small-world properties^37^. Small-world networks are widespread in social networks^37,40,45^ and have already been identified in FRDD populations on Torres Strait Islands, Australia^33^. They promote the rapid spread of diseases^38,45^. In addition, networks in our study are characterized by an over-dispersed and right-skewed degree distribution. Targeting a small number of highly-connected nodes in such networks can dramatically reduce the network connectivity, and its potential for diseases spread^46,47^. Therefore, in the case of rabies, targeting highly connected dogs in the identified networks would make vaccination campaigns more effective than random vaccination.

Another similarity detected between the study regions was that dogs in rural study sites tended to be more connected (higher relative degree and higher network density) than dogs in urban and semi-urban sites. There are several possible reasons for this, including smaller dog individual home range size or lower dog population density in urban settings. If individual home range size is a predictor for contact rates, dogs having smaller home ranges potentially have smaller degrees, as it has been suggested in another study^27^. In turn, smaller home range sizes may be caused by higher likelihood of dogs being confined, along with better supervision, better feeding practices, higher food availability, or more barriers for movements, such as big roads^26^. Previous studies on FRDD management, performed in various regions of the world, highlighted that dogs in urban settings are more frequently confined than dogs living in rural areas, for example because houses were better equipped with fences to prevent dogs from unsupervised roaming^48–51^. Responsible dog ownership, which refers to confinement practices but also identification, neutering and vaccination, is a critical point for rabies control^42,50,52^, but often impossible to be implemented by dog owners in rabies endemic countries by their own means. Therefore, measures supporting dog owners to reduce the home range size of their dogs, such as improving fenced building constructions, promoting feeding dogs at home and proper waste disposal, and offering neutering campaigns, are essential aspects of holistic rabies control programs. The influence of dog population density in the different study sites on the connectedness of the dogs could not be explored, since FRDD density data were not available from all study sites. It has been suggested that dog density is higher in urban areas, which would contradict the hypothesis that higher density leads to higher connectedness. However, since the confinement of dogs was also found to be more frequent in urban settings^48–51^, the dog density might be higher in urban areas but the number of dogs allowed to roam freely might be lower.

The MRQAP analysis revealed that the distance between owners' households was always negatively associated with the odds of a contact between two dogs. This implies that the dog networks are spatially driven, i.e. the closer two households are located, the higher the chance that the dogs living in those households will be in contact. This finding is consistent with a study performed in N'Djaména, the capital city of Chad^26^ and in Aboriginal communities in Northern Australia^41^. In N'Djaména, the analysis of FRDD contact networks resulted in the detection of closely connected network communities amongst dogs living close together^26^. If dog home range size is not influenced by dog density (as concluded from a FRDD study in Australia^30^), dogs would be more connected, and therefore disease may spread faster, in areas where dog density is high. Therefore, focusing control programs to high dog dense areas may have larger effects than in sparsely dog populated sites. Moreover, as distance between households influence contact patterns between dogs, the characteristics of the environment (e.g. type of land use, proximity to roads, potential physical barriers) between households and how FRDD select available environmental resources appears to be highly relevant in understanding dog's roaming behavior^53^.

Apart from detected similarities discussed before, the dog-level MRQAP and PBLM analysis revealed heterogeneity in the set of predictors for dogs' centrality and likelihood of a contact. What makes a dog central in the network, or influence the likelihood of a contact between two dogs, differs from one study site to another. This suggests that the local context matters. Male or female dogs, shepherd or hunting dogs might be managed differently from one country to another. For example, in the study site rural 2 in Chad, some interviewees reported that having a female dog was perceived as a potential source of problem because many people keep intact male as guardian dogs and the presence of a female in oestrus would result in dog fighting. This has not been reported in the other study sites, neither in the study site rural 2 in Chad. Despite the absence of dog management data to support this hypothesis, we can assume that if dogs are perceived differently across regions based on their sex, males and females might also be managed differently across regions. Similarly, dogs used for the same purpose may be managed differently from one country to another. For example in Australia, hunting dogs from several communities are gathered for pig hunting and therefore come into close contact between each other^54^—a practice that has not been reported in our study sites.

The local context is probably even more pronounced regarding the owner-level factors. Owner characteristics were investigated to explore if the owners’ characteristics and networks could influence the dog network. As all dogs in this study are owned, they might have a close relationship with their owner, follow them, and be more regularly in contact with the dogs of their owners' social circle. Moreover, the management of a dog may be impacted by the socio-economic status of its owner. Significant association found between owners' socioeconomic status and the likelihood of a contact between their dogs (e.g. higher odds for contacts between households with similar wealth level or the same ethnicity in Indonesia and Guatemala) is likely to reflect the owner social network. People with similar socioeconomic status might be more connected and so could their dogs. However, this is highly impacted by the owner–dog relationship and perception of the dog by the owner. Some people might walk with their dog around the village and take them during their daily trips, others prefer to keep them at home, regardless of the role of the dog. Perception of dogs varies between cultural groups^55^ and might impact the dog contact network in a way that has not been captured by the current study. Research would be need to understand the human–dog bound across various cultures and how it influences the social behavior of the dogs. Studies in India have highlighted dog's behavioral plasticity in interactions with humans^56–58^. They showed that dogs adapt their behavior towards humans depending on whether they are solitary or part of a group^59^, but information on how dog–human bounds impact dog sociability with conspecifics is lacking. In addition, studies on human mediated transportation networks might be of high value. Dog are impacted by human-induced movements^60^ and it might be easier to identify highly connected owners than highly connected dogs. Studies on dog transportation conducted in Africa identified long-range movements as a key factor for rabies persistence^61^.

The main objective of the study was to explore tangible factors that could be used during vaccination campaigns to target specific dogs. Therefore, we did not investigate some behavioural traits that are commonly reported as drivers of social network structures. Such factors include dominance rank, social style and reproduction state. Although these factors are likely to have an impact on dog's centrality, they cannot be easily assessed by an external person. Whether a female dog was in oestrus, based on the owner observation, was recorded during the questionnaire survey, to account for female reproductive state. However, the number of females observed to be in oestrus was very low (only 4% of female dogs). We hypothesized that FRDD owners might have less opportunity to observe their dogs and therefore do not always know their reproduction state; the reason why we did not consider this variable of uncertain quality in the analysis. The social style or dominance rank of a dog might influence its centrality in the network. More friendly dogs or dogs with high dominance rank may experience more frequent contacts. Dog personality and individuality have been studied for decades using approaches similar to human personality studies^62^. Tools were developed to characterize dog personality, to investigate dog behavior stability over time, studying dog personality traits in relation to owner (e.g. personality, demographics) and dog characteristics (e.g. breed, age), and to identify genetic factors associated to them^62–66^. This suggests that other factors than the one studied here are responsible for dog personality, which may have contributed to the heterogeneity we detected in our study. In addition, it would be interesting to assess the impact of resource distribution. Even if all dogs were daily fed by their owner, their nutritional needs may still be incomplete and dogs were likely to meet in places where they can find food, such as garbage dumps, markets or restaurants^67,68^.

In conclusion, we suggest that investigating the importance of social and cultural structures impacting owners and therefore shaping dog ecology would be needed to assess the potential use of targeted vaccination.

In this study, we decided not to exclude any contact data based on value of the received signal strength indicator (RSSI), such as done in earlier studies^26^. The rules applied there (monitoring contacts of less than 0.25 m only by excluding contacts with RSSI below – 75 dBm) seemed too restrictive for the purpose of our study. As each signal received by a contact sensor indicates that another dog was located at a maximum of 2 to 4 m away, we considered this as a contact. This is supported by a study in Australia where dog-borne video camera were used to investigate the nature of contact between two FRDD. It was found that most (69%) dog-to-dog interactions recorded by the camera included physical contact^68^. As dogs located in a 0 to 4 m range could to be visible on a camera, most of the contacts recorded by the contact sensors are likely to involve physical interactions. In addition, RSSI and distance do not have a linear relationship for distances above 0.25 m^26,69^. Therefore, by excluding signals with RSSI below – 75 dBm, contacts of less than a dog body length could have been excluded. However, to evaluate if the analysis restricted to close contacts (i.e. below 0.25 m) would affect our findings, the dog-level PBLM and MRQAP were repeated with excluding proximity events with an RSSI below – 75 dBm. The results slightly differed but the impact of the variables and conclusions were the same as for the full contact dataset (Supplementary Tables S28–S35 and Supplementary Figs. S5–S7).

We also evaluated the effect of more than one dog living in the same household on the results of the MRQAP. For example, if people own dogs of the same sex, those dogs will be in contact, not because they are of the same sex, but because they live together. Therefore, we developed a modified *netlogit* function that allows excluding contacts from dogs living in the same household from the analysis. The results modified *netlogit* function were found to be very similar to those obtained using the regular *netlogit* function (Supplementary Tables S36–S37).

Several limitations can be identified for the study. First, despite visiting the households several times, we were not able to collar all dogs in the study sites. The contact networks were therefore incomplete (Supplementary Methods). We do not consider that the missing dogs systematically biased our results since there is no evidence that dogs fearful to strangers are more or less connected. However, because we found a small proportion of dogs being highly connected, we might have missed some of these, which could have impacted the identification of the variables associated with the centrality measures. Second, the duration of the observation period was relatively short, limited by the battery capacity. As studies on owned FRDD contact networks are scare, the number of days needed to capture representative contact data is unknown. The duration of our study period lays in the range of other studies investigating contacts networks of FRDD on Torres Strait Islands and in N'Djaména^26,33^. This limit might have impacted our results to an unknown extend and could potentially be one of the reasons why common predictors were not identified. Longer study periods could allow us to investigate whether the centrality of a given dog varies over time. In addition, since our study design included several study populations that were studies only once, we could not assess whether the season influenced contact patterns. Different from wildlife, FRDD—still controlled by humans—do not have clear mating and pup rearing seasons, although the cycles are not evenly distributed over the years, according to a study from India^70^. A longitudinal study would therefore be of interest, to investigate possible changes in dog contact networks over time and assess the impact of the season, weather and temperature on contact structure variation, as it was found to have an influence on FRDD home range size^30^. Third, we did not analyze networks weighted by the time two dogs spend together, i.e. we did not differentiate between dogs spending most of the time versus dogs spending a few minutes per day together. From an epidemiological point of view, with the unweighted network analysis, we explored networks for spread of diseases that can be transmitted by a first, close contact, such as rabies. However, with the probability of rabies transmission given a bite being around 50%^71^, the frequency and duration of contact impacts the likelihood of rabies transmission, which may potentially change the findings of our study. It would be of interest to model disease transmission in our networks based on weighted networks, where weight corresponds to the time two dogs spend together. A fourth limitation of our study is related to the potential impact of rabies infection on dog behavior. Although changes of the dog contact network may explain sustainability of rabies within dog population^72^, knowledge of the impact on dog social behavior for rabid dogs remains very limited, because of the limitation to conduct such studies in the field. Therefore, the aim of the study was to investigate normal dog behavior. In this study, we did not explore the nature of a contact between two dogs, which could impact the likelihood of disease transmission. Bombara et al. in 2017 showed that even when contacts do not appear as aggressive, multiple teeth baring incidents could be observed during play fight, which could lead to rabies transmission^68^. We do not think excluding sick dogs impacted our results, as they do not represent rabid dog behavior nor normal dog behavior.

## Material and methods

### Study design and ethical approval

In each of the four countries, three study sites were selected—an urban or semi-urban and two rural—except in Chad, where two rural sites were selected. The selection of the sites was based on their expected number of dogs (Supplementary Table S38). The data were collected between January 2018 and January 2019. In each site, an area of one-kilometer square was defined using Google Earth. We aimed at including all owned domestic dogs whose owner’s household was located in these areas. Were included dogs that can roaming freely for at least part of the time, as well as dogs chained up, but still have the possibility to come in contact with free roaming dogs (with the exception of the urban site in Guatemala, where the dog density was very high and we focused on non-restrained dogs for capacity reasons). Dogs younger than four months old, which were too small to wear the collar, sick dogs and pregnant bitches (to avoid miscarriage due to stress) were excluded. To improve completeness of study population, each households was visited up to three time if the owner or the dog was absent. The study was presented to the head of the household or an adult person living in the same house. An informed consent was obtained from all dog owners who participated in the study. Recruited dog were offered to be vaccinated against rabies and/or dewormed. Very few owners refused to participate (reasons for not participating in Supplementary Methods).

Ethical approval was requested in each country: in Guatemala, it was granted by the Universidad del Valle de Guatemala (UVG) International Animal Care and Use Committee (Protocol No. I-2018(3)), the Ethics Review Board of the Committee for Research on Human Subjects of the Center for Health Studies in UVG (Protocol No. 175-04-2018) and by the Community Development Councils of the two rural areas (as it included Maya Q’eqchi’ communities); in Indonesia, it was granted by the Animal Ethics Commission of the Faculty of Veterinary Medicine, Nusa Cendana University (Protocol KEH/FKH/NPEH/2019/009); in Uganda, the study was accredited by the Uganda National Council for Science and Technology (Protocol NS640); in Chad, the National Chadian Bioethics Committee asserted that no formal ethical approval was needed for the study according to the Chadian regulation. Human and/or animal ethical approval were obtained depending on the country-specific regulations. All the procedures were carried out in accordance with relevant guidelines.

### Data collection

In total, 714 dogs (Table 1) were collared for 3–5 days (restricted by the battery capacity) with a geo-located contact sensor (GCS) that have earlier been used in another FRDD study^26^. The GCS was developed by Bonsai System (https://www.bonsai-systems.com/). It included a global positioning system (GPS) device and a contact sensor, functioning by Ultra-High Frequency Technology. The contact sensor recorded proximity to other contact sensors by broadcasting beacons every minute and constantly scanning for other sensors' beacons. The GCS were controlled by an application developed on iOS which allowed the user to start and stop the recording, download and erase the collected data using Bluetooth Low Energy.

Structured questionnaires were administered in each household to collect information on the collared dogs and their owners. The questionnaires were adapted to each country. Dog-level information included sex, age, BCS of the dog, purpose for keeping the dog (watch dog, shepherd dog, hunting dog, pet or meat production) and the period the dog was allowed to roam freely (permanently, all day, all night, a few hours, never). Owner-level information focused on their socioeconomic status, including ethnicity, religion, education level, professional occupation and income (Indonesia) or wealth related questions (other countries). The latter were assessed based on owner’s stated belongings, such as vehicle (e.g. car, motorbike, bike), livestock (e.g. cattle, pig, poultry) and other belongings (e.g. television, fridge, cellphone). The interviews were performed in local language by trained local team members. The data were electronically recorded using KoboCollect Android application (https://www.kobotoolbox.org/). The GPS location of each participating household was recorded.

### Statistical analysis

#### Social network analysis of the dog contact networks

A contact between two dogs was defined as a proximity event recorded by at least one of the two contact sensors, with no restriction based on the received RSSI applied. Network size (number of nodes), largest component size, network density, average shortest path length, clustering coefficient, degree and betweenness distribution were computed for each network (Supplementary Table S1). Skewness index ranging between 0.5–1 and above 1 indicate moderately and highly skewed distributions, respectively. A coefficient of variation higher than 50% indicates over-dispersion of the distribution. Unpaired Wilcoxon tests were used to assess if the distribution of those metrics differed between any two networks. To assess if the contact network displayed small-world properties, the average shortest path length and the clustering coefficient of the observed networks were compared with those of 1000 simulated random networks with the same number of nodes and edges as the largest component. The *p* value of the clustering coefficient (and average path length) was computed as the percentage of random networks for which the clustering coefficient was equal to or higher (and equal to or lower) than the observed empirical network. The small-world index is the ratio of the observed clustering coefficient to the mean clustering coefficient of the simulated random networks, divided by the ratio of the observed average shortest path to the mean average shortest path of the simulated random networks^37,72^.

To evaluate whether the results would differ if only close contacts would have been considered, 11 additional networks (i.e. one per study site) were generated with a contact defined as the recording of at least one signal, whose RSSI was above – 75 dBm, by at least one of the devices. According to static tests on the devices, all contacts closer than 25 cm are registered when RSSI is above this threshold^26^.

### Permutation-based linear regression models and quadratic assignment procedures

Log-transformed degree and betweenness were used as outcome variables for the PBLMs. This model type was selected because the assumption of independency between observations of standard linear regression models is violated with network data (i.e. a node's centrality depends on the centrality of its “neighbors”), increasing the risk of type I error^73^. As permutation-based models including random effect are not commonly available, separate models were implemented for each study site, which in turn enabled us to compare findings between study sites. The *p* values associated with each coefficient was computed based on permutation of the regression residuals. Information for the independent variables were directly retrieved from the questionnaires. To categorize wealth level and allocate owners in Chad, Guatemala and Uganda, where income information was not available, a hierarchical clustering based on a multiple factor analysis (MFA) was performed (Supplementary Methods, Supplementary Tables S5–S9, and Supplementary Fig. S3). The distance of the dog's home from the centroid of the study site (i.e. the centroid of the minimum convex polygon including all the household locations) was calculated using QGIS software. To respect the small size of the ten networks and the high number of explanatory variables, a loop was coded that selected the best models based on Akaike information criterion (AIC). Within the loop, PBLM were fitted for every possible combination of explanatory variables for each network (including the null model) and the AIC was calculated using the formula presented by Gordon^74^. The set of best models were identified for each network by including the model with the lowest AIC and all models with ΔAIC < 2 compared to the lowest AIC (see example for one network Supplementary Table S39). The variables' coefficients, their *p* values associated, and deviance explained by each explanatory variable included in these models^75^ were extracted for each model.

MRQAP were performed at the dog- and household-level. In there, multivariate logistic regression is applied to fit the occurrence of an edge in each dyad as the outcome of interest (Supplementary Table S22–S23). The Dekker's “semi-partialling plus” procedure was used, which is known to be robust to multicollinearity and autocorrelation^76^. Distance between households was computed using QGIS.

R statistical software was used to conduct the PBLM using *ape* package and *lmorigin* function^77,78^. The MFA was conducted with *FactoMineR* package, using *MFA* and *hcpc* functions^79^. The MRQAP was performed using the *sna* package and *netlogit* function^80^.

## Supplementary Information

Supplementary Information 1.

Supplementary Information 2.

Supplementary Information 3.

Supplementary Information 4.

Supplementary Information 5.

Supplementary Information 6.

Supplementary Information 7.

Supplementary Information 8.

Supplementary Information 9.
