# Supplementary material for: Predictors of free-roaming domestic dogs' contact network centrality and their relevance for rabies control
Source: Sci Rep. 2021 Jun 18;11:12898. doi: 10.1038/s41598-021-92308-7 (PMC8213792; doi:10.1038/s41598-021-92308-7)
Supplement: Supplementary file 1 — Supplementary Information 1. [file 41598_2021_92308_MOESM1_ESM.pdf]

Supplementary Information for

Predictors of free-roaming domestic dogs' contact network centrality and their relevance for rabies control

Charlotte Warembourg*, Guillaume Fournié, Mahamat Fayiz Abakar, Danilo Alvarez, Monica Berger-González, Terence Odoch, Ewaldus Wera, Grace Alobo, Elfrida Triasny Ludvina Carvallo, Valentin Dingamnayal Bal, Alexis Leonel López Hernandez, Enos Madaye, Filipe Maximiano Sousa, Abakar Naminou, Pablo Roquel, Sonja Hartnack, Jakob Zinsstag, Salome Dürr

Supplementary Methods

Reasons for not participating

Reasons for not participating in the study included the absence of the dog, the absence of the owner or the person who can handle the dog, the dog showing high level of stress or aggressiveness, dogs being too small or too large for wearing the collars, refusal of the owner to participate (beliefs (e.g. taking blood would be detrimental for the dog and could lead to the dog's sickness or death), refusal to participate in research projects in general, concerns that the collar would bother the dog while hunting, owners did not have their ID cards (necessary in Guatemala for filling the written consent form), and lack of interest in the study or in rabies vaccination.

Wealth characterization: Multiple Factor Analysis

The objective of the Multiple Factor Analysis (MFA) and hierarchical clustering analysis were to construct wealth categories for countries where owner's income was not available (i.e. Chad, Uganda, Guatemala). These categories aimed at being explanatory variables in the household-level models (i.e. household-level PBLM and household-level MRQAP). Information was collected during the owner's interview using a questionnaire (Questionnaire S1-S4). To fit the countries' particular socioeconomic situation, different sets of variables were used in each country (Table 1). For example, in Chad, information about personal belongings (e.g. television, oven) were useful to investigate wealth in settled populations. However, mobile pastoralists, because of their lifestyle, own very few personal belongings. Livestock is a strong indicator of economic wealth and the main source of income in those communities [1].

Multiple factor analysis

A MFA was conducted to generate synthetic variables accounting for most of the variability in the set of wealth economic variables, which included both continuous and categorical variables (Supplementary Table S5), except in Sinetaye (Rural 2 – Chad), where the households only differed on a group of continuous variables and a principal component analysis was performed. The

questionnaire forms are available as supplementary material. The MFA was performed at the country level, except for Chad where the analysis at the country level fully separated the owners by study site. Therefore, to categorize the wealth within our two study areas in Chad, a MFA was performed separately for each study site (Rural 1 and rural 2).
The number of components included in the analysis was selected based on a visual basis of the eigenvalues histogram to explain the variability of the data with as few dimensions as possible (Supplementary Figure S3). The number of components was set to two for all the countries, explaining in total 56.1%, 71.4%, 22.8% and 31% of the variance for Chad-rural 1, Chad-rural 2, Indonesia and Uganda, respectively.

Hierarchical clustering analysis
The number of clusters selected through hierarchical clustering analysis (HCA) is the one with the higher relative loss of inertia, with the inertia being the sum of within- and between-class inertia. The selected partition is the one for which an additional merge between clusters leads to a high loss in inertia between clusters[2]. A cluster can be constituted of a single household or groups of households.
The HC resulted in the creation of three clusters in each study area (Supplementary Tables S6-S9).
The MFA and the hierarchical clustering was performed using the *FactoMineR* package in R software[32].

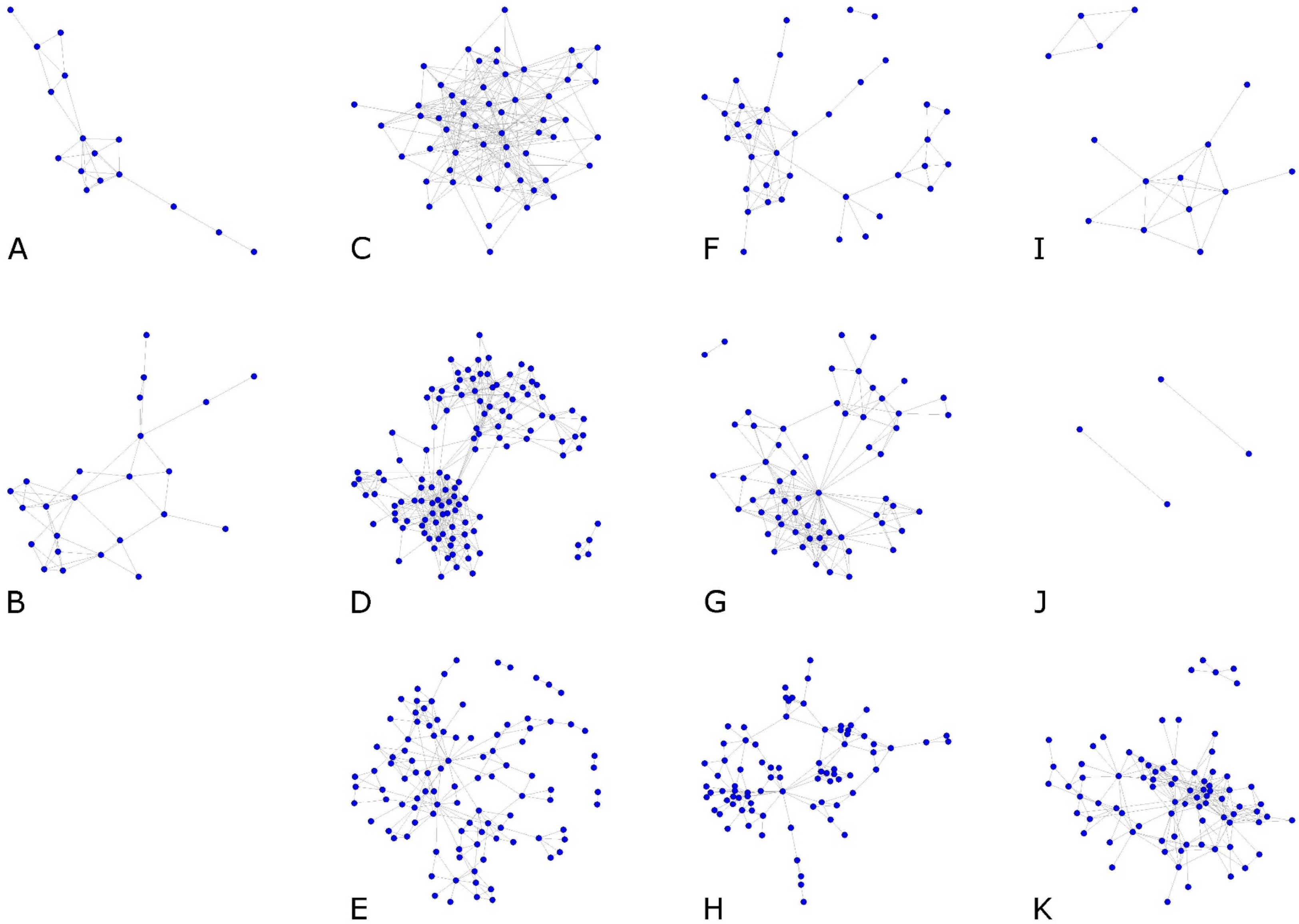

Supplementary Fig. S1. Dog contact networks of all 11 study sites. Nodes represent dogs and links contacts between two dogs. A. Chad - rural 1, B. Chad – rural 2, C. Guatemala - rural 1, D. Guatemala, rural 2, E. Guatemala, urban/semi-urban, F. Indonesia - rural 1, G. Indonesia – rural 2, H. Indonesia – urban-semi-urban, I. Uganda – rural 1, J. Uganda – rural 2 (not further analysed), K. Uganda - urban/semi-urban.

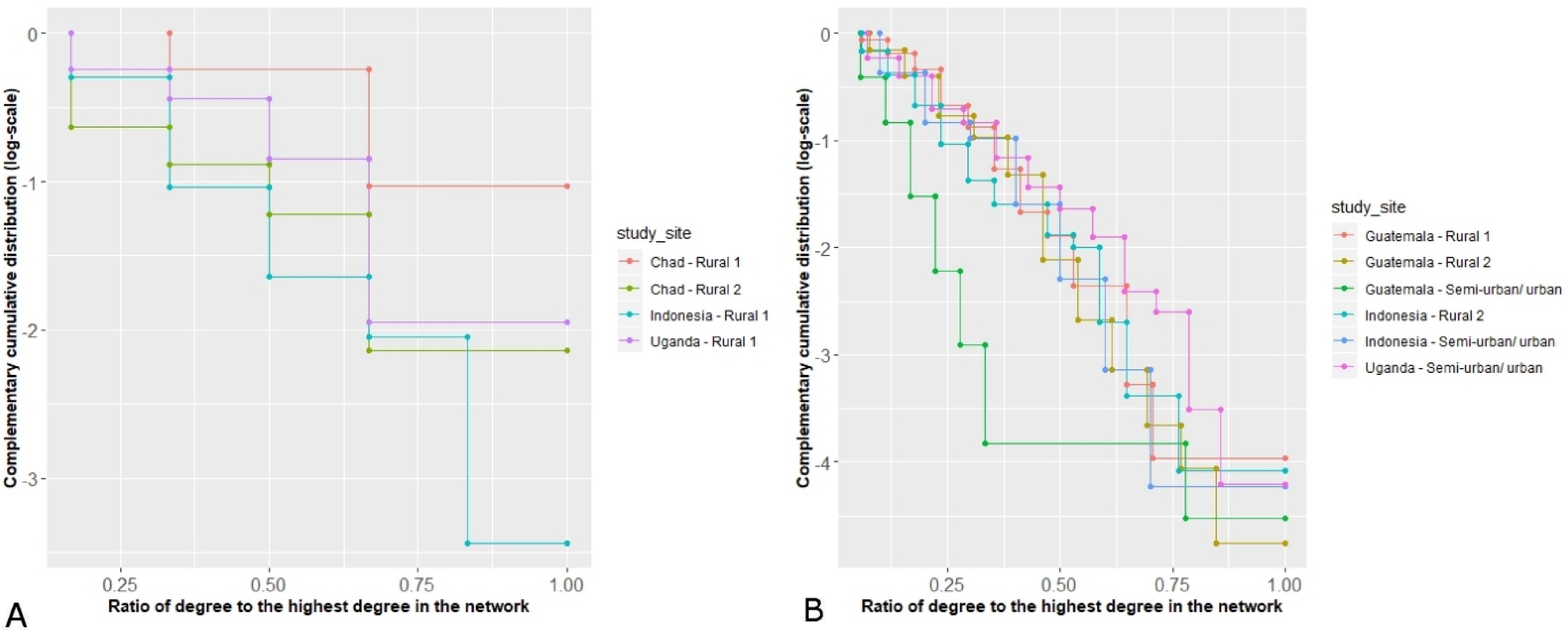

Supplementary Fig. S2. Cumulative degree distribution of network constituted of (A) less and (B) more than 50 dogs on a linear-log scale. For comparison purposes between the networks, the degree was divided by the highest degree of each network in the cumulative distribution. A straight line indicates that the degree distribution display an exponential decay.

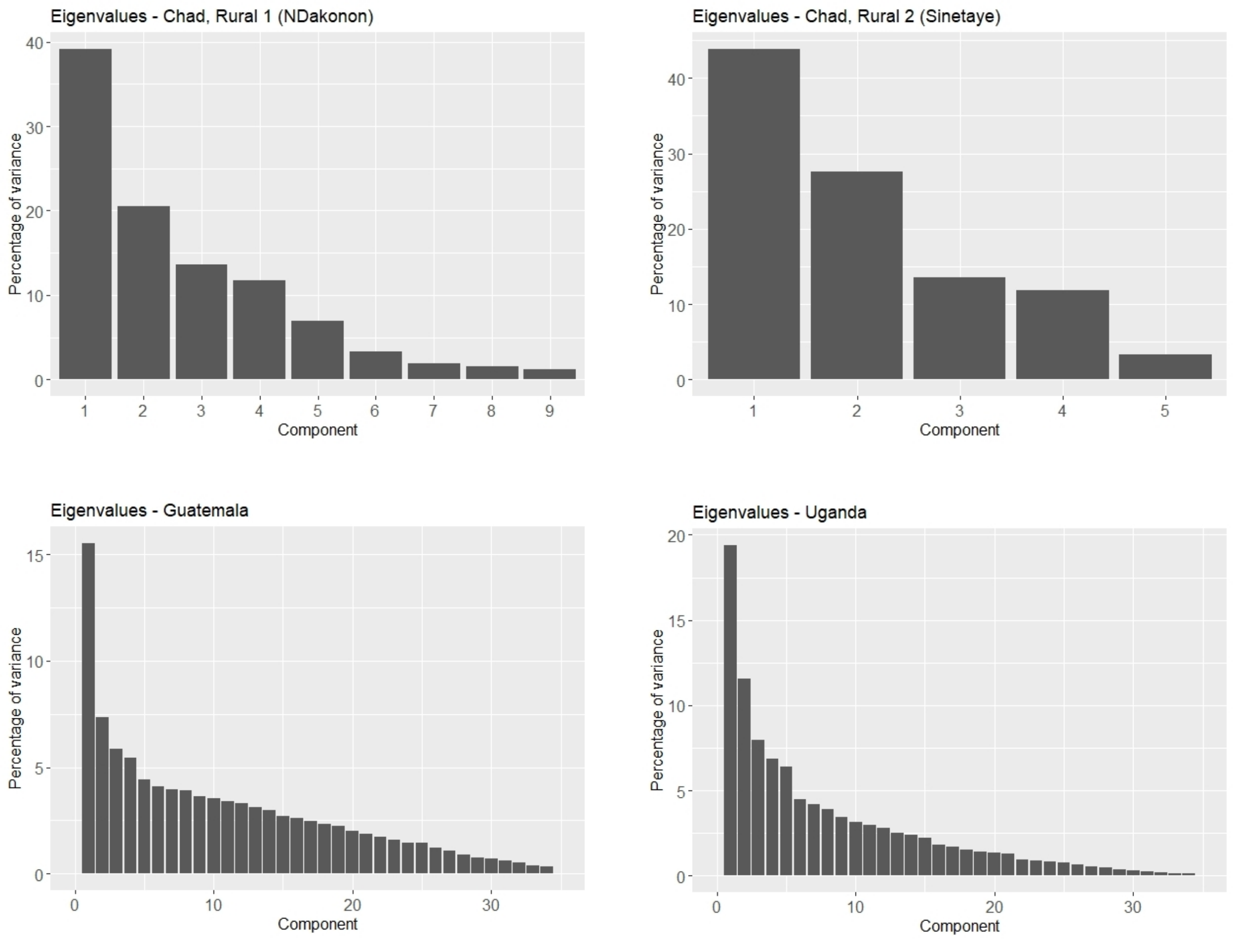

Supplementary Fig. S3. Percentage of variance explained by each component of the MFA (Chad (Rural 1), Guatemala and Uganda) or PCA (Chad (Rural 2)). Two components were included in the analysis.

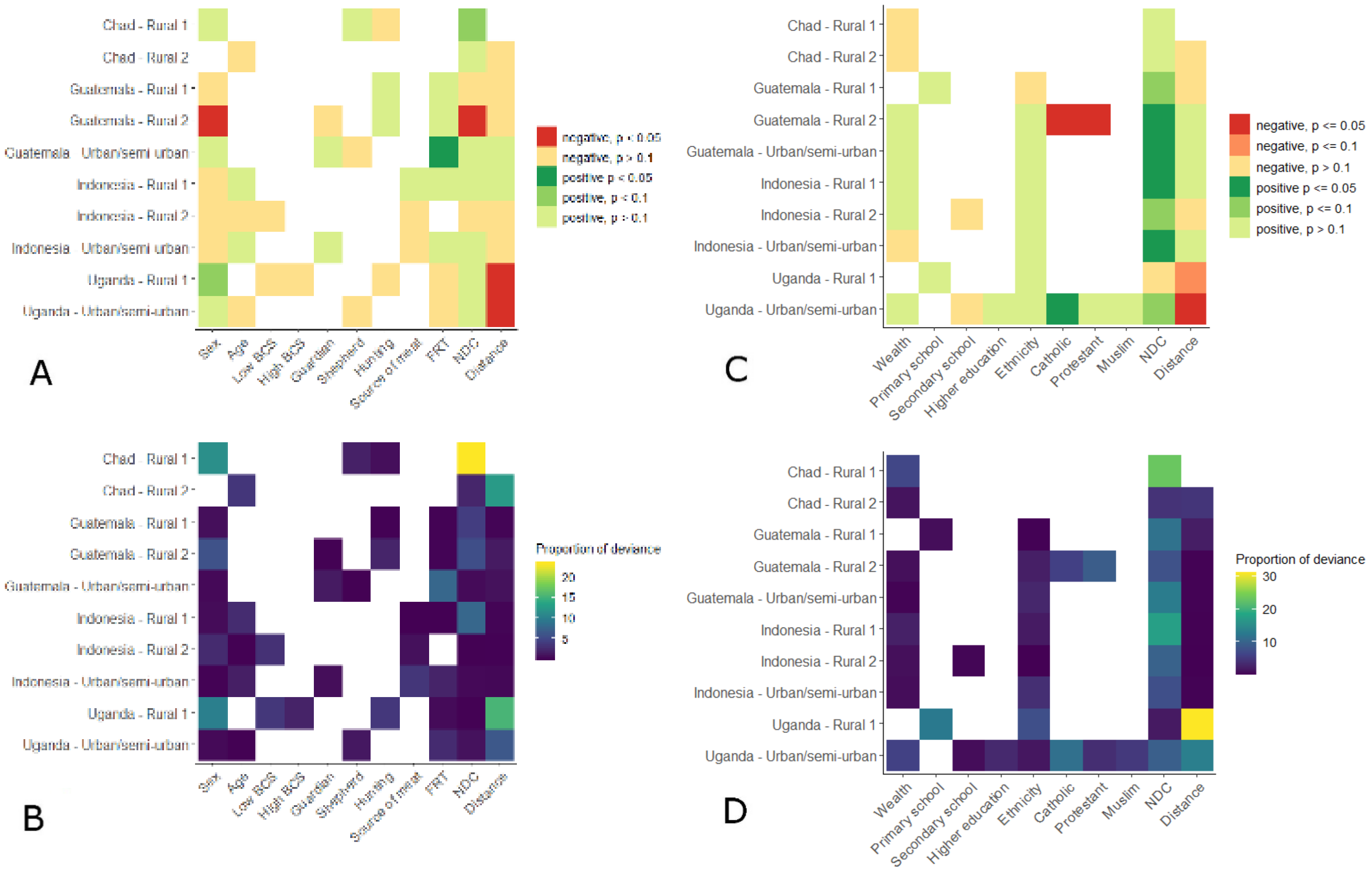

Supplementary Fig. S4. Permutation-based multivariate linear regression model results with betweenness as output. A. Significance and coefficient sign of the dog-level network. B: Proportion of deviance explained by each variable of the dog-level network. C. Significance and coefficient sign of the household-level network. D. Proportion of deviance explained by each variable of the household-level network. Empty field denote variables that were not explored or where not selected for the best models by the PBLM.

Dog-level factors: dog's sex (Sex – 0: male (baseline), 1: female); body conditioning score (BCS) of 2 and lower (Low BCS), BCS of 4 and higher (High BCS) with the baseline of BCS = 3; being a guardian dog (Guardian =1, dummy variable), hunting dog (Hunting, dummy variable), shepherd dog (Shepherd, dummy variable) or raised for meat (Source of meat, dummy variable); free-roaming time (FRT – range from 0 to 10); number of dogs collared per household (NDC, contiguous variable) and distance per 100 meters from dogs home to the centroid of the study site (Distance, continuous variable).
Household-level factors: wealth category based on the Multiple Factor Analysis or the income when available (Wealth, with the lowest level (i.e. poorest) being the baseline); owner finalizing primary school (Primary school), finalizing secondary school (Secondary school), finalizing professional training or university (Higher Education), with absence of formal education being the baseline; belonging to the main local ethnicity (Ethnicity, dummy variable), being catholic (Catholic, dummy variable), being evangelic (Evangelic, dummy variable); number of dogs collared per household (NDC, contiguous variable) and distance per 100 meters from household to the centroid of the study area (Distance, continuous variable).

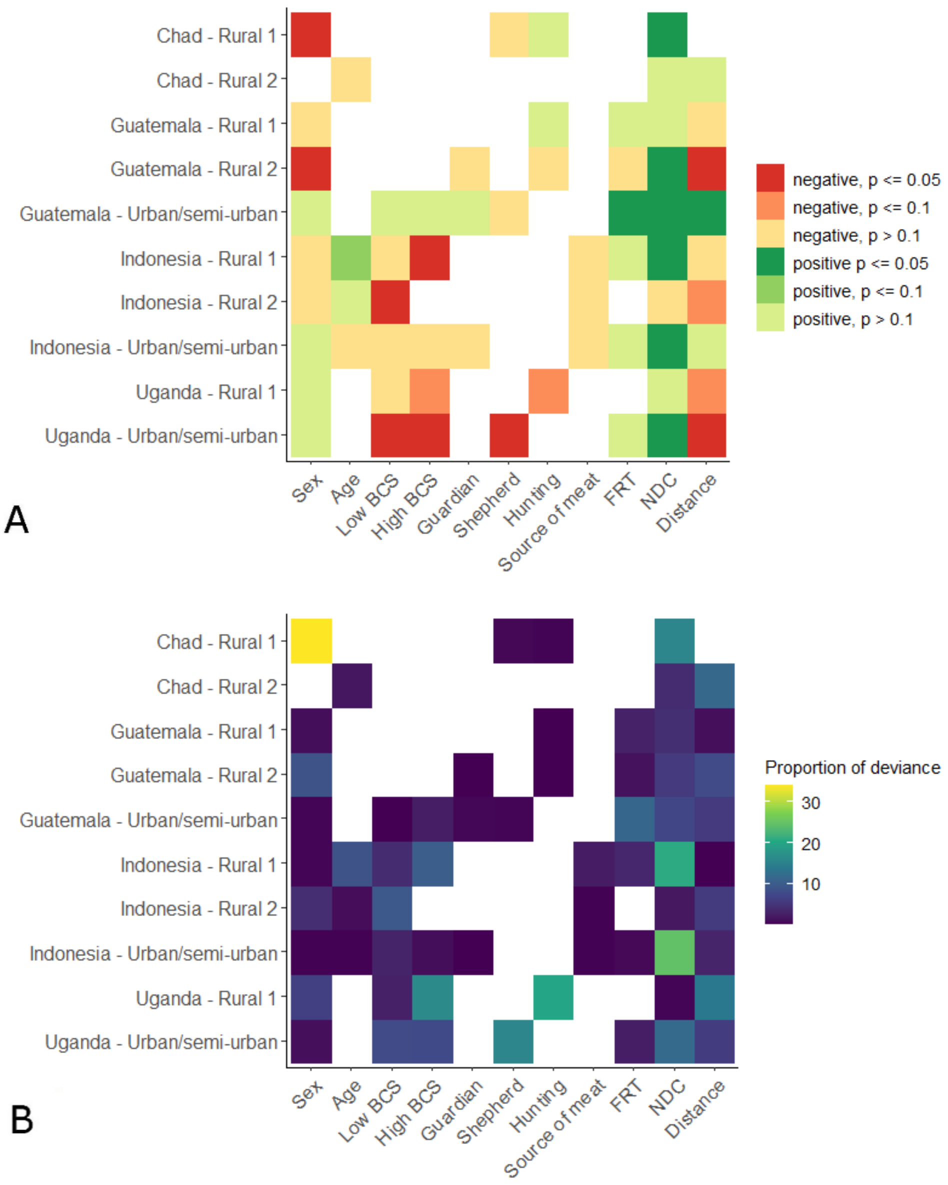

Supplementary Fig. S5. Dog- level permutation-based linear model results with degree as output, excluding the proximity events with a RSSI below -75dBm. A. Significance and coefficient sign. B: Proportion of deviance explained by each variable. Empty field denote variables that were not explored or where not selected for the best models by the MRQAP.

Dog-level factors: dog's sex (Sex – 0: male (baseline), 1: female); body conditioning score (BCS) of 2 and lower (Low BCS), BCS of 4 and higher (High BCS) with the baseline of BCS = 3; being a guardian dog (Guardian =1, dummy variable), hunting dog (Hunting, dummy variable), shepherd dog (Shepherd, dummy variable) or raised for meat (Source of meat, dummy variable); free-roaming time (FRT – range from 0 to 10); number of dogs collared (NDC, contiguous variable) and distance per 100 meters from dogs home to the centroid of the study site (Distance, continuous variable).

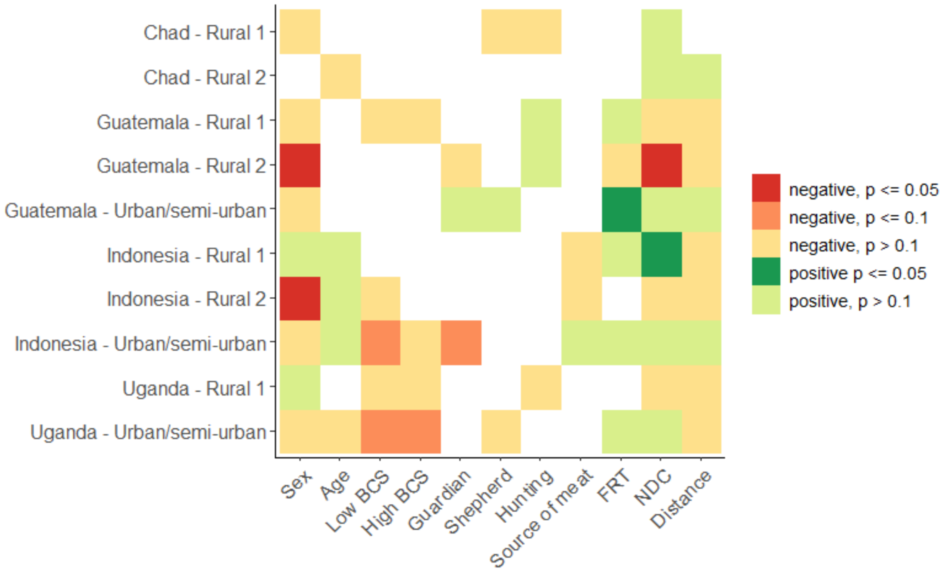

A

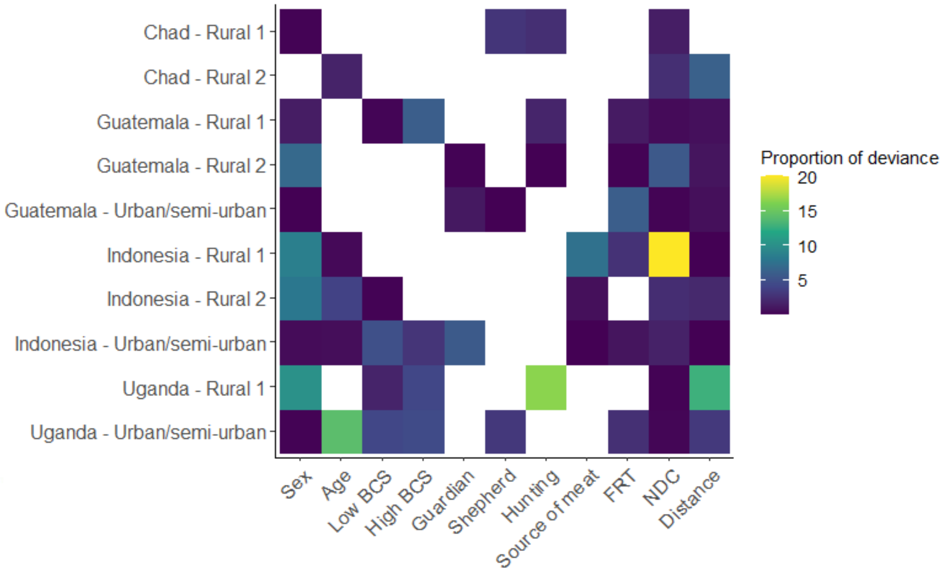

B

Supplementary Fig. S6. Dog- level permutation-based linear model results with betweenness as output, excluding the proximity events with a RSSI below -75dBm. A. Significance and coefficient sign. B: Proportion of deviance explained by each variable. Empty field denote variables that were not explored or where not selected for the best models by the PBLM.

Dog-level factors: dog's sex (Sex – 0: male (baseline), 1: female); body conditioning score (BCS) of 2 and lower (Low BCS), BCS of 4 and higher (High BCS) with the baseline of BCS = 3; being a guardian dog (Guardian =1, dummy variable), hunting dog (Hunting, dummy variable), shepherd dog (Shepherd, dummy variable) or raised for meat (Source of meat, dummy variable); free-roaming time (FRT – range from 0 to 10); number of dogs collared (NDC, contiguous variable) and distance per 100 meters from dogs home to the centroid of the study site (Distance, continuous variable).

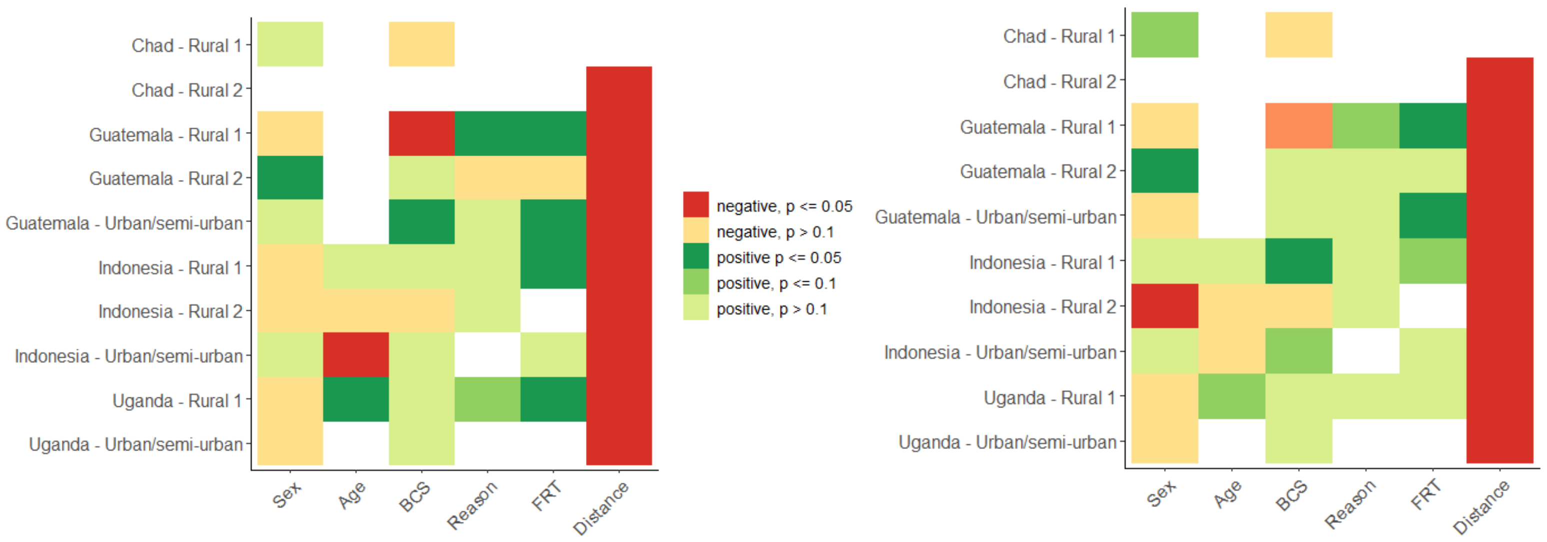

Supplementary Fig. S7. Odds ratios derived from the multiple regression quadratic assignment procedure for having a contact between dogs, using the modified *netlogit* function (A) or excluding the proximity events with a RSSI below -75dBm (B) having the same level per variable investigated. Positive: OR > 1, negative OR < 1. Empty field denote variables that were not explored or where not selected for the best models by the MRQAP.

Dog-level factors: same sex (male versus female), same age category (more or less than two years old), same BCS category (more or less than 2), same reason for keeping the dog (guardian, hunting, shepherd, source of meat), similar free-roaming time (FRT, always free-roaming, free-roaming by day, by night, a few hours per day or never) and distance per 100 meters between the households (Distance, continuous variable). Household-level factors: same wealth category (Wealth, cluster 1 to 4), same education level (Education: no formal education, primary education, secondary education, higher education), same ethnicity (Ethnicity, various levels depending on the study site), same religion (Religion, various levels depending on the study site) and distance per 100 meters between the households (Distance, continuous variable).

Supplementary Table S1. Node- and network-level metrics

Metric	Description
Network-level metrics	
Network size	Number of connected dogs (i.e. number of dogs being in contact with at least one other dog in the network)*
Size of the largest component	Number of nodes in the largest component of the network, i.e. dogs who are directly or indirectly connected
Density	Number of observed edges over the number of possible edges in the network ignoring isolated nodes* (i.e. number of edges if all the dogs were in contact with each other)
Shortest path length between two nodes	Number of edges on the shortest path between these two nodes
Average shortest path length	Mean of the shortest path length between all pair of nodes of the largest component
Relative average shortest path	Average shortest path length divided by the size of the largest component
Clustering coefficient	Probability that adjacent nodes of a node in the largest component are connected
Node-level metrics	
Degree	Number of adjacent nodes to a given node
Betweenness	Number of times a node sits on the shortest path between two other nodes
Relative degree	Degree divided by the network size
Normalized betweenness	Betweenness divided by the maximum possible betweenness in the network. The normalized betweenness corresponds to the fraction of all possible shortest paths in the network on which a dog lie.

* We choose not to consider isolated nodes because it is impossible to differentiate truly isolated dogs (i.e. dogs that did not have contact with any other dog during the data collection period) and dogs of which their collar was defective.

Supplementary Table S2. Average shortest path length and clustering coefficient of the observed and simulated networks and small world index. For the average path length, the p-value corresponds to the proportion of simulated random networks having an average path length shorter than the observed average path length. For the clustering coefficient, the p-value corresponds to the proportion of simulated networks having a clustering coefficient larger than the observed clustering coefficient.

		**Chad**		**Guatemala**			**Indonesia**			**Uganda**		
		Rural 1	Rural 2	Rural 1	Rural 2	Urban/ semi-urban	Rural 1	Rural 2	Urban/ semi-urban	Rural 1	Rural 2	Urban/ semi-urban
Average shortest path length of the largest component		2.5	2.6	2.1	3.2	3.8	3.2	2.5	4.0	1.8	-	3.0
Average shortest path length of the largest component in simulated random networks	Mean (min - max)	2.1 (1.8 – 2.4)	2.3 (2.1 – 2.7)	1.98 (1.95-2.03)	2.6 (2.5 – 2.6)	3.6 (3.2 – 3.8)	2.5 (2.3 - 2.7)	2.3 (2.2 – 2.4)	3.1 (3.0 - 3.3)	2.1 (1.7 – 2.6)	-	2.5 (2.5 – 2.6)
	P-value	1	1	1	1	1	1	1	1	0.002	-	1
Clustering coefficient		0.56	0.45	0.39	0.38	0.27	0.5	0.46	0.42	0.58	-	0.43
Clustering coefficient of simulated random networks	Mean (min - max)	0.23 (0.03–0.45)	0.17 (0.04-0.31)	0.17 (0.13-0.21)	0.06 (0.05-0.09)	0.04 (0.01-0.08)	0.12 (0.05-0.22)	0.12 (0.09-0.17)	0.05 (0.02-0.11)	0.23 (0-0.45)	-	0.08 (0.05-0.12)
	P-value	0	0	0	0	0	0	0	0	0	-	0
Small world index		2.0	2.3	2.2	5.1	6.4	3.3	3.5	6.5	2.9	-	4.5

Supplementary Table S3. Coding of the dog-level permutation-based linear models' independent variables. Some of the factors presented here were investigated as part of another study on free-roaming domestic dog roaming behavior[4].

Variable	Type of variable	Coding (all study sites)
Sex	Binary	0: male<br>1: female
Age	Continuous	Age in months
Low BCS	Binary	0: BCS higher than 2<br>1: BCS lower or equal to 2
High BCS	Binary	0: BCS lower than 4<br>1: BCS higher or equal to 4
Guardian dog	Binary	0: not a guardian dog<br>1: guardian dog
Shepherd dog	Binary	0: not a shepherd dog<br>1: shepherd dog
Hunting dog	Binary	0: not a hunting dog<br>1: hunting dog
Dog for meat source	Binary	0: not raised for meat<br>1: raised for meat
Free-roaming time	Count	0: never free-roaming<br>4: free-roaming a few hours per day<br>12: free roaming only by day or only by night<br>24: always free-roaming
Number of dogs collared	Count	Number of dogs collared in the same household
Distance	Continuous	Distance per 100 meters between the household and the centroid of the minimum convex polygon around all the participating households

Supplementary Table S4. Coding of the household-level permutation-based linear models' independent variables

Variable	Type of variable	Coding			
		**Chad**	**Guatemala**	**Uganda**	**Indonesia**
Wealthier according to the MFA wealth categories	Binary	Rural 1<br>0: cluster 1 and 2<br>1: cluster 3<br><br>Rural 2<br>0: cluster 1<br>1: cluster 2 and 3	0: cluster 2 and 3<br>1: cluster 1	0: cluster 1<br>1: cluster 2 and 3	-
Wealthier according to the monthly salary in Rupiah	Binary	-			Rural 1 and 2<br>0: < Rp 500.000<br>1: > Rp 500.000<br><br>Semi-urban<br>0: < Rp 1 million<br>1: > Rp 1 million
Primary school	Binary	0: finished primary school<br>1: did finished primary school			
Secondary school	Binary	0: finished secondary school<br>1: did finished secondary school			
Higher than secondary school	Binary	0: did not finished a university degree or professional training<br>1: finished a university degree or professional training			
Main ethnicity	Binary	0: do not belong to the main local ethnicity<br>1: below to the main local ethnicity			
Catholic	Binary	0: not catholic<br>1: catholic			
Protestant	Binary	0: not protestant<br>1: protestant			
Muslim	Binary	0: not Muslim<br>1: Muslim			
Distance	Continuous	Distance per 100 meters between the household and the centroid of the minimum convex polygon around all the participating households			

Supplementary Table S5. Description of the groups used in the multiple factor analysis and principal component analysis per country.

Country	Group	Variables included in the group
Chad – NDakonon (Rural 1)	Travel	- The respondent traveled outside of the village/nomad camp during the last 2 years (binary)
	Commodities	- The household has drinkable water (binary)<br>- The household has electricity (binary)
	Belongings	- The respondent possesses a fridge (binary)<br>- The respondent possesses radio (binary)<br>- The respondent possesses television (binary)<br>- The respondent possesses cellphone (binary)
	Transportation	- The respondent owns a bike (binary)<br>- The respondent owns a car (binary)<br>- The respondent owns a motorbike (binary)
	Livestock	- Total number of cattle owned by the respondent (count)<br>- Total number of goats owned by the respondent (count)<br>- Total number of sheep owned by the respondent (count)<br>- Total number of camels owned by the respondent (count)<br>- Total number of horses or donkeys owned by the respondent (count)<br>- Total number of poultry owned by the respondent (count)
Chad – Sinetaye (Rural 2)	Livestock	- Total number of cattle owned by the respondent (count)<br>- Total number of goats owned by the respondent (count)<br>- Total number of sheep owned by the respondent (count)<br>- Total number of camels owned by the respondent (count)<br>- Total number of horses or donkeys owned by the respondent (count)<br>- Total number of poultry owned by the respondent (count)
Guatemala	Profession	- Profession of the respondent (categorical - levels: farmer, construction worker, seller, housewife, student, house worker, housemaid, agricultural worker, professional, other)
	House	- The house is made of block (binary)<br>- The house is made of wood (binary)<br>- The house is made of cob (binary)<br>- The house is made of palm (binary)<br>- The house is made of metal sheet (binary)<br>- Presence of foundation (binary)
	Commodities	- The household has electricity (binary)<br>- The household has drinkable water (binary)<br>- The household has a bathroom (binary)<br>- The household has latrines (binary)<br>- The household has solar panels (binary)
	Belongings	- The respondent possesses a gas cocker (binary)<br>- The respondent possesses a fridge (binary)<br>- The respondent possesses a radio (binary)<br>- The respondent possesses a television (binary)<br>- The respondent possesses a microwaves (binary)
	Transportation	- The respondent owns a bike (binary)<br>- The respondent owns a car (binary)<br>- The respondent owns a motorbike (binary)<br>- The respondent owns a tuk-tuk (binary)<br>- The respondent owns a truck (binary)<br>- The respondent does not own any mean of transportation (binary)

Uganda	Profession	- Profession of the respondent (categorical, levels: crop farming, livestock keeping, salaried employment, casual laboring, boda-boda (motorcycle) driver, charcoal burning, poultry keeping, bee keeping, trade, other)
	House	- Roof material (categorical, levels: block/brick (concrete), block (mud), wood, mud and wood, other)<br>- Walls material (categorical, levels: iron sheet, grass thatch, tiles, other)<br>- Floor material (categorical, levels: soil, cement, ceramic floor (tiles))<br>- Presence of foundation (binary)
	Commodities	- The household has electricity (binary)<br>- The household has a bathroom (binary)<br>- The household has toilets (binary)<br>- The household has pit latrines (binary)<br>- The household has none of the items above (binary)
	Source of water	- The main source of water for the household is rain water (binary)<br>- The main source of water for the household is tape water (binary)<br>- The main source of water for the household is water from a bore hole (binary)<br>- The main source of water for the household is water from the national supply (NWSC) (binary)
	Transportation	- The respondent owns a bike (binary)<br>- The respondent owns a car (binary)<br>- The respondent owns a motorbike (binary)<br>- The respondent owns a truck (binary)<br>- The respondent to not own any mean of transportation (binary)
	Livestock	- Total number of cattle owned by the respondent (binary)<br>- Total number of goats owned by the respondent (binary)<br>- Total number of sheep owned by the respondent (binary)<br>- Total number of pigs owned by the respondent (binary)<br>- Total number of poultry owned by the respondent (binary)<br>- Total number of beehives owned by the respondent (binary)

Supplementary Table S6. Characteristics of the clusters resulting from hierarchical clustering in Chad, rural 1 (NDakonon)

Cluster	1	2	3
Number of households	3	12	1
Characteristics of the cluster	The owners of this cluster raise a higher number of poultry, they do not have drinkable water nor television	The owners of this cluster raise a lower number of poultry, they do not have drinkable water nor television	The owner in this cluster is the only person having both drinkable water and a television.

Supplementary Table S7. Characteristics of the clusters resulting from hierarchical clustering in Chad, rural 2 (Sinetaye)

Cluster	1	2	3
Number of households	14	1	8
Characteristics of the cluster	The owners do not raise any camel but a few cattle, goat and sheep	The owner of this cluster is the only person raising camels	The owners do not raise any camel but raise more cattle, goat and sheep than the first cluster.

Supplementary Table S8. Characteristics of the clusters resulting from hierarchical clustering in Guatemala

Cluster	1	3	4
Number of households	71	49	49
Characteristics of the cluster	The owners in this cluster are mostly sellers, students or professionals. Their house walls are in block and wood, the soil is in cement, granite or ceramic. All the houses with foundations are in this cluster. Nearly everybody has electricity and water but no solar panels. All the trucks owning households are in this cluster. Some people own cars and more than half of the owners have bikes and motorbikes	The owners in this cluster are mostly farmers. Some people are construction workers. Their houses are in wood, palm and block. The floor is made of soil or cement. Most of the people have electricity and half of them have drinkable water. There are a few bathroom and many toilets. The main mean of transportation are bikes and motorbikes.	The owners in this cluster are mostly farmers and housewives. Most of the houses are built with wood, the roof is in palm leaves and the floor is made of soil. Few people have drinkable water and electricity but more people have solar panels compared to the other clusters. They own a few bikes and motorbikes but most of them do not have any personal mean of transportation.

Supplementary Table S9. Characteristics of the clusters resulting from hierarchical clustering in Uganda

Cluster	1	2	3
Number of households	20	36	16
Characteristics of the cluster	The owners in this cluster have various job and various mean of transportation. The house walls are built in concrete, iron sheet, cement or ceramic. Nearly all household have electricity bathroom, toilets and latrines. They own some livestock. The water usually comes from national supply and tape water	The owners in this cluster have various job and various mean of transportation but a third of the owners do not have any mean of transportation. The house is made of concrete, iron sheet, cement. Unlike cluster 1, they have no ceramic floor. Most of the people have electricity, toilets, and latrines. They own a few livestock. Drinkable water comes from national supply and tape water	The owners in this cluster are mostly farmers and livestock keepers. Most of them have bikes. The house are made of mud, grass thatch and soil. They barely have no commodities. Water often comes from a borehole. They own some livestock

Supplementary Table S10. Regression coefficient of the dog-level PBLM using log-transformed degree as output. Green: positive coefficient, yellow: null coefficient, red: negative coefficient

Country	Study area	Sex	Age	Low BCS	High BCS	Guardian	Shepherd	Hunting	Source of meat	FRT	NDC	Distance
Chad	Rural 1	-0.45					0.54	-0.20			0.25	
Chad	Rural 2		0.00								0.31	-0.16
Guatemala	Rural 1	-0.13						0.04		0.01	0.01	-0.08
Guatemala	Rural 2	-0.31				-0.27		0.01		-0.01	0.01	-0.03
Guatemala	Urban/ semi-urban	0.06		-0.10	0.24	0.22	-0.16			0.02	0.15	0.05
Indonesia	Rural 1	0.14	0.01						-0.24	-0.03	0.59	-0.13
Indonesia	Rural 2	-0.05	0.00	-0.43					-0.30		-0.14	-0.17
Indonesia	Urban/ semi-urban	0.08	0.00	-0.36	-0.31	0.16			-0.10	0.02	0.22	0.00
Uganda	Rural 1	0.52		-0.19	-0.93			-0.54		-0.01	0.03	-0.18
Uganda	Urban/ semi-urban	0.11	0.00				-1.16			-0.02	0.14	-0.20

Supplementary Table S11. Significance of the dog-level PBLM coefficients using degree as output. Black: p<0.05, grey: p<0.1, light grey: p>0.1

Country	Study area	Sex	Age	Low BCS	High BCS	Guardian	Shepherd	Hunting	Source of meat	FRT	NDC	Distance
Chad	Rural 1	0.12					0.26	0.74			0.1	
Chad	Rural 2		0.58								0.44	0.53
Guatemala	Rural 1	0.5						0.84		0.22	0.82	0.32
Guatemala	Rural 2	0.01				0.28		0.94		0.16	0.8	0.42
Guatemala	Urban/ semi-urban	0.66		0.46	0.29	0.22	0.72			0	0.02	0.27
Indonesia	Rural 1	0.62	0.25						0.66	0.36	0.01	0.21
Indonesia	Rural 2	0.82	0.65	0.09					0.24		0.31	0.18
Indonesia	Urban/ semi-urban	0.64	0.9	0.06	0.34	0.69			0.63	0.05	0.01	0.98
Uganda	Rural 1	0.1		0.48	0.01			0.07		0.5	0.92	0.04
Uganda	Urban/ semi-urban	0.6	0.57				0			0.55	0.01	0.02

Supplementary Table S12. Proportion of deviance explained the each variable of the dog-level PBLM using log-transformed degree as output. Purple: low, yellow: high

Country	Study area	Sex	Age	Low BCS	High BCS	Guardian	Shepherd	Hunting	Source of meat	FRT	NDC	Distance
Chad	Rural 1	13.18					8.67	0.46			14.82	
Chad	Rural 2		1.51								2.82	1.89
Guatemala	Rural 1	0.97						0.08		3.12	0.11	2.10
Guatemala	Rural 2	5.49				0.97		0.00		1.69	0.05	0.57
Guatemala	Urban/ semi-urban	0.19		0.50	1.02	1.44	0.12			10.04	5.39	1.15
Indonesia	Rural 1	0.67	3.52						0.27	2.39	23.27	4.21
Indonesia	Rural 2	0.10	0.35	5.14					2.45		1.83	3.22
Indonesia	Urban/ semi-urban	0.25	0.02	4.16	1.05	0.20			0.27	4.45	9.78	0.00
Uganda	Rural 1	3.80		0.49	18.31			6.79		0.34	0.01	11.25
Uganda	Urban/ semi-urban	0.37	0.42				11.10			0.48	8.87	6.92

Supplementary Table S13. Regression coefficient of the household-level PBLM using log-transformed degree as output. Green: positive coefficient, yellow: null coefficient, red: negative coefficient

Country	Study area	Wealth	Primary school	Secondary school	Higher education	Ethnicity	Catholic	Protestant	NDC	Distance
Chad	Rural 1	-0.77							0.34	
Chad	Rural 2	-0.01							0.45	-0.15
Guatemala	Rural 1		0.03			-0.22			0.16	-0.15
Guatemala	Rural 2	0.03				0.14	-0.31	-0.43	0.23	0.00
Guatemala	Urban/ semi-urban	0.11				0.64	0.62	0.78	0.22	0.01
Indonesia	Rural 1	0.11	-0.96	-1.05	-0.64	0.09			0.59	-0.19
Indonesia	Rural 2	-0.19		-0.01		0.41			0.16	-0.10
Indonesia	Urban/ semi-urban	0.01				0.42			0.28	-0.03
Uganda	Rural 1		0.31			0.09			-0.11	-0.25
Uganda	Urban/ semi-urban	0.45		-0.19	0.38	0.04			0.11	-0.27

Supplementary Table S14. Significance of the household-level PBLM coefficients using log-transformed degree as output. Black: p<0.05, grey: p<0.1, light grey: p>0.1

Country	Study area	Wealth	Primary school	Secondary school	Higher education	Ethnicity	Catholic	Protestant	NDC	Distance
Chad	Rural 1	0.3							0.14	
Chad	Rural 2	0.98							0.48	0.61
Guatemala	Rural 1		0.92			0.43			0.08	0.08
Guatemala	Rural 2	0.86				0.31	0.21	0.08	0	0.97
Guatemala	Urban/ semi-urban	0.66				0.11	0.25	0.14	0.04	0.88
Indonesia	Rural 1	0.62	0.12	0.09	0.43	0.74			0.01	0.04
Indonesia	Rural 2	0.45		0.97		0.07			0.24	0.4
Indonesia	Urban/ semi-urban	0.94				0.03			0.04	0.6
Uganda	Rural 1		0.51			0.87			0.61	0.01
Uganda	Urban/ semi-urban	0.07		0.51	0.31	0.9			0.09	0.01

Supplementary Table S15. Proportion of deviance explained the each variable of the household-level PBLM using log-transformed degree as output. Purple: low, yellow: high

Country	Study area	Wealth	Primary school	Secondary school	Higher education	Ethnicity	Catholic	Protestant	NDC	Distance
Chad	Rural 1	12.88							17.66	
Chad	Rural 2	0.00							3.04	1.53
Guatemala	Rural 1		0.03			1.92			9.72	9.85
Guatemala	Rural 2	0.04				1.30	2.03	4.08	11.74	0.00
Guatemala	Urban/ semi-urban	0.33				4.21	2.16	3.55	6.95	0.04
Indonesia	Rural 1	0.64	6.34	7.21	2.13	0.32			20.95	11.26
Indonesia	Rural 2	1.59		0.01		10.34			4.09	1.91
Indonesia	Urban/ semi-urban	0.01				8.38			8.54	0.52
Uganda	Rural 1		1.61			0.14			1.29	48.64
Uganda	Urban/ semi-urban	7.74		0.98	2.38	0.04			7.20	18.68

Supplementary Table S16. Regression coefficient of the dog-level PBLM using log-transformed betweenness as output. Green: positive coefficient, yellow: null coefficient, red: negative coefficient

Country	Study area	Sex	Age	Low BCS	High BCS	Guardian	Shepherd	Hunting	Source of meat	FRT	NDC	Distance
Chad	Rural 1	1.58					0.97	-0.84			1.15	
Chad	Rural 2		-0.02								0.88	-1.33
Guatemala	Rural 1	-0.33						0.10		0.01	-0.26	-0.07
Guatemala	Rural 2	-1.21				-0.22		1.11		0.01	-0.54	-0.19
Guatemala	Urban/ semi-urban	0.36				0.98	-0.32			0.08	0.20	0.19
Indonesia	Rural 1	-0.34	0.02						0.20	0.00	0.96	0.08
Indonesia	Rural 2	-0.81	0.00	-1.00					-0.49		-0.06	-0.07
Indonesia	Urban/ semi-urban	-0.22	0.03			1.20			-1.52	0.05	0.19	-0.12
Uganda	Rural 1	2.08		-1.29	-0.75			-0.98		-0.04	0.14	-0.53
Uganda	Urban/ semi-urban	0.33	0.00				-1.12			-0.12	0.11	-0.54

Supplementary Table S17. Significance of the dog-level PBLM coefficients using log-transformed betweenness as output. Black: p<0.05, grey: p<0.1, light grey: p>0.1

Country	Study area	Sex	Age	Low BCS	High BCS	Guardian	Shepherd	Hunting	Source of meat	FRT	NDC	Distance
Chad	Rural 1	0.17					0.6	0.78			0.06	NA
Chad	Rural 2		0.37								0.48	0.1
Guatemala	Rural 1	0.58						0.88		0.84	0.16	0.77
Guatemala	Rural 2	0.01				0.82		0.11		0.67	0.01	0.2
Guatemala	Urban/ semi-urban	0.57				0.26	0.88			0.01	0.51	0.38
Indonesia	Rural 1	0.71	0.4						0.92	1	0.14	0.8
Indonesia	Rural 2	0.25	0.9	0.22					0.55		0.89	0.85
Indonesia	Urban/ semi-urban	0.77	0.29			0.53			0.12	0.23	0.61	0.64
Uganda	Rural 1	0.06		0.2	0.32			0.19		0.6	0.88	0.04
Uganda	Urban/ semi-urban	0.6	0.9				0.38			0.18	0.45	0.04

Supplementary Table S18. Proportion of deviance explained the each variable of the dog-level PBLM using log-transformed betweenness as output. Purple: low, yellow: high

Country	Study area	Sex	Age	Low BCS	High BCS	Guardian	Shepherd	Hunting	Source of meat	FRT	NDC	Distance
Chad	Rural 1	11.55					1.76	0.63			23.24	
Chad	Rural 2		3.50								2.16	12.56
Guatemala	Rural 1	0.64						0.05		0.08	4.04	0.17
Guatemala	Rural 2	5.87				0.04		2.05		0.15	5.54	1.35
Guatemala	Urban/ semi-urban	0.35				1.36	0.03			7.76	0.46	0.80
Indonesia	Rural 1	0.49	2.64						0.02	0.00	7.74	0.21
Indonesia	Rural 2	2.55	0.04	3.01					0.73		0.04	0.07
Indonesia	Urban/ semi-urban	0.11	1.63			0.58			3.44	2.00	0.36	0.30
Uganda	Rural 1	10.01		3.51	1.91			3.56		0.46	0.04	15.36
Uganda	Urban/ semi-urban	0.41	0.03				1.19			2.58	0.81	6.45

Supplementary Table S19. Regression coefficient of the household-level PBLM using log-transformed betweenness as output. Green: positive coefficient, yellow: null coefficient, red: negative coefficient

Country	Study area	Wealth	Primary school	Secondary school	Higher education	Ethnicity	Catholic	Protestant	Muslim	NDC	Distance
Chad	Rural 1	-1.99								1.39	
Chad	Rural 2	-0.46								1.45	-0.72
Guatemala	Rural 1		0.39			-0.13				0.54	-0.22
Guatemala	Rural 2	0.42				0.53	-1.55	-1.89		0.58	0.04
Guatemala	Urban/ semi-urban	0.33				2.12				1.20	0.07
Indonesia	Rural 1	0.82				0.78				2.01	0.05
Indonesia	Rural 2	0.36		-0.27		0.00				0.73	-0.03
Indonesia	Urban/ semi-urban	-0.54				1.30				1.20	0.07
Uganda	Rural 1		1.75			1.45				-0.27	-0.41
Uganda	Urban/ semi-urban	1.23		-0.53	1.36	0.81	2.91	1.43	2.48	0.41	-0.71

Supplementary Table S20. Significance of the household-level PBLM coefficients using log-transformed betweenness as output. Black: p<0.05, grey: p<0.1, light grey: p>0.1

Country	Study area	Wealth	Primary school	Secondary school	Higher education	Ethnicity	Catholic	Protestant	Muslim	NDC	Distance
Chad	Rural 1	0.63								0.18	
Chad	Rural 2	0.63								0.36	0.35
Guatemala	Rural 1		0.62			0.87				0.06	0.4
Guatemala	Rural 2	0.39				0.18	0.03	0.01		0.01	0.78
Guatemala	Urban/ semi-urban	0.72				0.16				0	0.77
Indonesia	Rural 1	0.4				0.47				0.03	0.92
Indonesia	Rural 2	0.59		0.75		1				0.09	0.95
Indonesia	Urban/ semi-urban	0.55				0.16				0.05	0.82
Uganda	Rural 1		0.2			0.25				0.65	0.09
Uganda	Urban/ semi-urban	0.13		0.57	0.25	0.43	0.03	0.21	0.15	0.06	0.03

Supplementary Table S21. Proportion of deviance explained the each variable of the dog-level PBLM using log-transformed betweenness as output. Purple: low, yellow: high.

Country	Study area	Wealth	Primary school	Secondary school	Higher education	Ethnicity	Catholic	Protestant	Muslim	NDC	Distance
Chad	Rural 1	6.64								23.44	
Chad	Rural 2	1.26								4.36	4.54
Guatemala	Rural 1		0.78			0.08				11.74	2.16
Guatemala	Rural 2	0.92				2.21	5.86	9.22		7.93	0.10
Guatemala	Urban/ semi-urban	0.19				3.03				13.11	0.12
Indonesia	Rural 1	2.41				1.86				17.62	0.05
Indonesia	Rural 2	0.92		0.29		0.00				9.80	0.02
Indonesia	Urban/ semi-urban	0.71				3.92				7.55	0.10
Uganda	Rural 1		12.65			7.54				1.78	31.33
Uganda	Urban/ semi-urban	5.88		0.76	3.20	1.52	11.56	3.79	4.99	9.50	13.40

Supplementary Table S22. Coding of the dog-level multiple quadratic regression assignment procedure variables

Variable	Modalities	Type of variable	Coding (all study sites)
Sex	Male, female	Binary	0: opposite sex<br>1: same sex
Age	Less than 2 years, 2 years and more	Binary	0: same age category<br>1: different age category
Body condition score (BCS)	2 and less, 3 and more	Binary	0: same BCS category<br>1: different BCS category
Role of the dog (multiple answers possible)	Guardian, shepherd, hunting, source of meat	Binary	0: same role<br>1: different role
Free-roaming time		Count	0: dogs are never free-roaming at the same time<br>0.5: both dogs are roaming a few hours<br>1: one dog is roaming on daytime (or at night-time) and the other dog is roaming a few hours<br>2: one dog is always free-roaming and the other dog is roaming a few hours<br>5: one dog is always free-roaming and the second is free-roaming during the day only (respectively the night)<br>10: dogs are free-roaming at the same time (i.e. always, per day or per night)
Distance		Continuous	Distance per 100 meters between the owner's households

Supplementary Table S23. Coding of the household-level multiple quadratic regression assignment procedure variables

Variable	Levels	Type of variable	Coding			
			Chad	Guatemala	Uganda	Indonesia
Wealth	Cluster 1 to 3	Binary	0: different wealth category<br>1: same wealth category			
Income (montly salary in Rupiah)	< Rp 500.000, Rp 500.000 - 1.000.000, Rp 1.000.000 - 2.000.00, > Rp 2.000.000	Binary	-			1: same income level
Education	No formal education, primary school, secondary school, higher than secondary school	Binary	0: different education level<br>1: same education level			
Ethnicity	Varies between countries	Binary	0: different ethnicity<br>1: same ethnicity			
Religion	Varies between countries	Binary	O: different religion<br>1: same religion			
Distance	Distance	Continuous	Distance per 100 meters between the owner's households			

Supplementary Table S24. Odds ratio of the dog-level MRQAP. Green: positive coefficient, yellow: null coefficient, red: negative coefficient

Country	Study area	Sex	Age	BCS	Reason	FRT	Distance
Chad	NDakonon	1.22		0.62			
Chad	Sinetaye						0.26
Guatemala	Poptun	0.88		0.67	1.83	1.09	0.42
Guatemala	Sabaneta	1.47		1.05	0.98	0.99	0.39
Guatemala	Romana	1.02		1.57	1.52	1.10	0.45
Indonesia	Habi	0.97	0.89	1.43	1.36	1.11	0.38
Indonesia	Hepang	0.77	0.73	0.91	1.58		0.43
Indonesia	Pogon	1.04	0.38	1.72		1.04	0.36
Uganda	Soroti	1.00	1.58	1.40	2.14	1.06	0.48
Uganda	Kamuda1	0.46		1.28			0.52

Supplementary Table S25. Significance of the dog-level MRQAP. Black: p<0.05, grey: p<0.1, light grey: p>0.1

Country	Study area	Sex	Age	BCS	Reason	FRT	Distance
Chad	NDakonon	0.59		0.43			
Chad	Sinetaye						0.00
Guatemala	Poptun	0.48		0.03	0.03	0.00	0.00
Guatemala	Sabaneta	0.01		0.68	0.94	0.77	0.00
Guatemala	Romana	0.92		0.01	0.22	0.03	0.00
Indonesia	Habi	0.90	0.83	0.20	0.32	0.01	0.00
Indonesia	Hepang	0.18	0.43	0.79	0.21		0.00
Indonesia	Pogon	0.92	0.04	0.11		0.67	0.00
Uganda	Soroti	0.99	0.02	0.22	0.11	0.10	0.00
Uganda	Kamuda1	0.46		0.87			0.00

Supplementary Table S26. Odds ratio of the household-level MRQAP. Green: positive coefficient, yellow: null coefficient, red: negative coefficient

Country	Study area	Wealth	Education	Ethnicity	Religion	Distance
Chad	NDakonon	2.22	0.30			
Chad	Sinetaye	0.86				0.30
Guatemala	Poptun	1.02	1.24	0.60	1.37	0.60
Guatemala	Sabaneta	1.65	0.95	1.39	0.98	0.66
Guatemala	Romana		0.78	0.65	0.69	0.55
Indonesia	Habi	0.91	1.38	1.16		0.43
Indonesia	Hepang	1.07	0.84	1.49		0.38
Indonesia	Pogon	0.76	0.94	1.00		0.44
Uganda	Soroti	0.97	0.71	0.61	1.19	0.71
Uganda	Kamuda1		6.04	0.89	0.83	0.66

Supplementary Table S27. Significance of the household-level MRQAP. Black: p<0.05, grey: p<0.1, light grey: p>0.1

Country	Study area	Wealth	Education	Ethnicity	Religion	Distance
Chad	NDakonon	0.37	0.17			
Chad	Sinetaye	0.74				0.00
Guatemala	Poptun	0.95	0.42	0.21	0.24	0.00
Guatemala	Sabaneta	0.02	0.84	0.02	0.90	0.00
Guatemala	Romana		0.30	0.27	0.13	0.00
Indonesia	Habi	0.75	0.27	0.65		0.00
Indonesia	Hepang	0.79	0.56	0.07		0.00
Indonesia	Pogon	0.48	0.90	1.00		0.00
Uganda	Soroti	0.92	0.31	0.15	0.53	0.00
Uganda	Kamuda1		0.14	0.88	0.91	0.01

Supplementary Table S28. Regression coefficient of the dog-level PBLM using log-transformed degree as output, excluding the proximity events with a RSSI below -75dBm. Green: positive coefficient, yellow: null coefficient, red: negative coefficient.

Country	Study area	Sex	Age	Low BCS	High BCS	Guardian	Shepherd	Hunting	Source of meat	FRT	NDC	Distance
Chad	Rural 1	-0.49					-0.09	0.08			0.17	
Chad	Rural 2		0.00								0.36	0.43
Guatemala	Rural 1	-0.15						0.03		0.02	0.10	-0.06
Guatemala	Rural 2	-0.43				-0.02		-0.03		-0.01	0.16	-0.13
Guatemala	Urban/ semi-urban	0.07		0.02	0.37	0.11	-0.21			0.02	0.17	0.11
Indonesia	Rural 1	-0.08	0.01	-0.26	-0.97				-0.47	0.02	0.42	-0.01
Indonesia	Rural 2	-0.32	0.00	-0.55					-0.06		-0.12	-0.21
Indonesia	Urban/ semi-urban	0.04	0.00	-0.27	-0.30	-0.05			-0.04	0.01	0.34	0.08
Uganda	Rural 1	0.58		-0.39	-0.91			-0.82			0.14	-0.18
Uganda	Urban/ semi-urban	0.18		-0.54	-1.26		-1.31			0.04	0.14	-0.18

Supplementary Table S29. Significance of the dog-level PBLM using degree as output, excluding the proximity events with a RSSI below -75dBm. Black: p<0.05, grey: p<0.1, light grey: p>0.1

Country	Study area	Sex	Age	Low BCS	High BCS	Guardian	Shepherd	Hunting	Source of meat	FRT	NDC	Distance
Chad	Rural 1	0					0.69	0.78			0.03	
Chad	Rural 2		0.65								0.45	0.18
Guatemala	Rural 1	0.52						0.91		0.27	0.16	0.5
Guatemala	Rural 2	0				0.94		0.9		0.21	0.01	0
Guatemala	Urban/ semi-urban	0.61		0.86	0.11	0.5	0.56			0	0	0.02
Indonesia	Rural 1	0.73	0.07	0.21	0.04				0.3	0.19	0.01	0.93
Indonesia	Rural 2	0.14	0.49	0.02					0.81		0.34	0.08
Indonesia	Urban/ semi-urban	0.79	0.76	0.12	0.38	0.94			0.84	0.49	0	0.11
Uganda	Rural 1	0.17		0.33	0.09			0.07			0.75	0.09
Uganda	Urban/ semi-urban	0.36		0.01	0.01		0			0.14	0	0.02

Supplementary Table S30. Proportion of deviance explained the each variable of the dog-level PBLM using log-transformed degree as output, excluding the proximity events with a RSSI below -75dBm. Purple: low, yellow: high.

Country	Study area	Sex	Age	Low BCS	High BCS	Guardian	Shepherd	Hunting	Source of meat	FRT	NDC	Distance
Chad	Rural 1	34.11					0.49	0.20			15.72	
Chad	Rural 2		1.66								3.72	11.49
Guatemala	Rural 1	0.89						0.03		2.70	4.26	0.92
Guatemala	Rural 2	8.93				0.00		0.02		1.24	5.57	7.75
Guatemala	Urban/ semi-urban	0.28		0.03	2.36	0.42	0.26			11.23	7.14	5.56
Indonesia	Rural 1	0.27	8.87	3.85	10.15				2.18	3.35	20.88	0.01
Indonesia	Rural 2	4.10	0.83	9.43					0.10		1.70	5.70
Indonesia	Urban/ semi-urban	0.08	0.11	2.93	0.91	0.01			0.05	0.57	24.65	3.08
Uganda	Rural 1	6.33		2.64	16.27			19.98			0.30	13.69
Uganda	Urban/ semi-urban	0.95		7.73	7.57		15.71			2.30	11.74	5.96

Supplementary Table S31. Regression coefficient of the dog-level PBLM using log-transformed betweenness as output, excluding the proximity events with a RSSI below -75dBm. Green: positive coefficient, yellow: null coefficient, red: negative coefficient.

Country	Study area	Sex	Age	Low BCS	High BCS	Guardian	Shepherd	Hunting	Source of meat	FRT	NDC	Distance
Chad	Rural 1	-0.04					-0.37	-0.48			0.09	
Chad	Rural 2		-0.01								0.82	0.88
Guatemala	Rural 1	-0.63		-0.23	-4.18			0.87		0.03	-0.11	-0.18
Guatemala	Rural 2	-1.81				-0.36		0.11		-0.01	-0.75	-0.21
Guatemala	Urban/ semi-urban	0.00				0.87	0.15			0.08	0.10	0.17
Indonesia	Rural 1	1.57	0.01						-3.53	0.09	1.63	-0.03
Indonesia	Rural 2	-1.70	0.02	-0.20					-0.55		-0.55	-0.46
Indonesia	Urban/ semi-urban	-0.43	0.01	-1.52	-2.74	-6.57			0.05	0.04	0.37	0.01
Uganda	Rural 1	1.79		-0.78	-1.12			-1.81			-0.18	-0.42
Uganda	Urban/ semi-urban	-0.21	-0.01	-1.80	-3.57		-0.37			0.18	0.07	-0.52

Supplementary Table S32. Significance of the dog-level PBLM using log-transformed betweenness as output, excluding the proximity events with a RSSI below -75dBm. Black: p<0.05, grey: p<0.1, light grey: p>0.1.

Country	Study area	Sex	Age	Low BCS	High BCS	Guardian	Shepherd	Hunting	Source of meat	FRT	NDC	Distance
Chad	Rural 1	0.88					0.64	0.4			0.55	
Chad	Rural 2		0.64								0.55	0.36
Guatemala	Rural 1	0.46		0.79	0.11			0.36		0.46	0.66	0.58
Guatemala	Rural 2	0.01				0.79		0.9		0.76	0.01	0.34
Guatemala	Urban/ semi-urban	1				0.33	0.94			0.02	0.77	0.5
Indonesia	Rural 1	0.12	0.76						0.16	0.42	0.02	0.94
Indonesia	Rural 2	0.03	0.13	0.83					0.56		0.26	0.26
Indonesia	Urban/ semi-urban	0.64	0.57	0.09	0.15	0.07			0.96	0.46	0.33	0.98
Uganda	Rural 1	0.39		0.68	0.55			0.27			0.92	0.29
Uganda	Urban/ semi-urban	0.82	0.5	0.09	0.08		0.82			0.2	0.7	0.13

Supplementary Table S33. Proportion of deviance explained the each variable of the dog-level PBLM using log-transformed betweenness as output, excluding the proximity events with a RSSI below -75dBm. Purple: low, yellow: high.

Country	Study area	Sex	Age	Low BCS	High BCS	Guardian	Shepherd	Hunting	Source of meat	FRT	NDC	Distance
Chad	Rural 1	0.07					2.95	2.49			1.37	
Chad	Rural 2		1.68								2.52	6.23
Guatemala	Rural 1	1.28		0.18	6.06			1.84		1.20	0.39	0.63
Guatemala	Rural 2	6.90				0.07		0.01		0.07	5.61	0.84
Guatemala	Urban/ semi-urban	0.00				1.08	0.01			6.04	0.11	0.60
Indonesia	Rural 1	8.65	0.33						7.54	2.71	20.15	0.02
Indonesia	Rural 2	7.96	3.90	0.08					0.59		2.37	2.05
Indonesia	Urban/ semi-urban	0.42	0.53	4.93	2.90	5.69			0.00	0.84	1.62	0.00
Uganda	Rural 1	10.16		1.77	4.22			16.56			0.08	12.85
Uganda	Urban/ semi-urban	0.08	14.22	4.28	4.56		3.13			2.55	0.19	3.21

Supplementary Table S34. Odds ratio of the dog-level MRQAP, excluding the proximity events with a RSSI below -75dBm. Green: positive coefficient, yellow: null coefficient, red: negative coefficient

Country	Study area	Sex	Age	BCS	Reason	FRT	Distance
Chad	NDakonon	2.84		0.61			
Chad	Sinetaye						0.35
Guatemala	Poptun	0.91		0.66	1.78	1.12	0.35
Guatemala	Sabaneta	1.70		1.14	1.51	1.03	0.34
Guatemala	Romana	0.95		1.34	1.65	1.09	0.43
Indonesia	Habi	1.14	1.00	2.33	1.58	1.11	0.30
Indonesia	Hepang	0.52	0.79	0.97	1.57		0.37
Indonesia	Pogon	1.42	0.49	2.28		1.22	0.37
Uganda	Soroti	1.00	1.68	1.72	2.53	1.08	0.25
Uganda	Kamuda1	0.45		2.12			0.43

Supplementary Table S35. Significance of the dog-level MRQAP, excluding the proximity events with a RSSI below -75dBm. Black: p<0.05, grey: p<0.1, light grey: p>0.1

Country	Study area	Sex	Age	BCS	Reason	FRT	Distance
Chad	NDakonon	0.07		0.20			
Chad	Sinetaye						0.00
Guatemala	Poptun	0.69		0.07	0.07	0.00	0.00
Guatemala	Sabaneta	0.00		0.40	0.23	0.14	0.00
Guatemala	Romana	0.84		0.15	0.15	0.05	0.00
Indonesia	Habi	0.66	1.00	0.02	0.27	0.05	0.00
Indonesia	Hepang	0.01	0.53	0.92	0.23		0.00
Indonesia	Pogon	0.47	0.19	0.08		0.13	0.00
Uganda	Soroti	0.99	0.09	0.15	0.32	0.18	0.00
Uganda	Kamuda1	0.51		0.53			0.01

Supplementary Table S36. Odds ratio of the dog-level MRQAP, using the modified *netlogit* function. Green: positive coefficient, yellow: null coefficient, red: negative coefficient

Country	Study area	Sex	Age	BCS	Reason	FRT	Distance
Chad	NDakonon	1.04		0.65			
Chad	Sinetaye						0.27
Guatemala	Poptun	0.83		0.58	1.89	1.09	0.51
Guatemala	Sabaneta	1.52		1.04	0.89	0.98	0.45
Guatemala	Romana	1.09		1.56	1.17	1.11	0.52
Indonesia	Habi	0.89	1.15	1.35	1.16	1.09	0.38
Indonesia	Hepang	0.78	0.73	0.94	1.48		0.44
Indonesia	Pogon	1.03	0.39	1.61		1.03	0.37
Uganda	Soroti	0.98	1.75	1.25	2.09	1.09	0.59
Uganda	Kamuda1	0.42		1.15			0.54

Supplementary Table S37. Significance of the dog-level MRQAP, using the modified *netlogit* function. Black: $p<0.05$, grey: $p<0.1$, light grey: $p>0.1$

Country	Study area	Sex	Age	BCS	Reason	FRT	Distance
Chad	NDakonon	0.89		0.42			
Chad	Sinetaye						0.00
Guatemala	Poptun	0.37		0.01	0.04	0.00	0.00
Guatemala	Sabaneta	0.00		0.78	0.64	0.22	0.00
Guatemala	Romana	0.66		0.01	0.58	0.00	0.00
Indonesia	Habi	0.57	0.80	0.23	0.56	0.01	0.00
Indonesia	Hepang	0.19	0.29	0.82	0.17		0.00
Indonesia	Pogon	0.92	0.03	0.16		0.61	0.00
Uganda	Soroti	0.92	0.01	0.38	0.05	0.03	0.00
Uganda	Kamuda1	0.41		0.89			0.00

Supplementary Table S38. Description of the study area and data collection, adapted from Warembourg et al.[4]

Country	Collaborative institute	Study area	Village/Town	Expected number of dogs	Study period	Localization	Description
Chad	Institut de Recherche en Élevage pour le Développement (IRED)	Rural 1	NDakonon	Low	Jan/Feb 2018	Region: Moyen-Chari Department: Grande Sido District: Danamadji	In both locations in Chad, the population is constituted of settled residents and mobile pastoralists. They are constituted of several families, living in concentration camps, while settled communities live in stable villages[5]. Mobiles pastoralists travel through transhumance routes during the wet season and stay near water points during the dry season. The main ethnic groups in Yao are Sara, Fulani, Goranes and Arabs, in addition to other more local populations. Settled communities are mainly agriculturalists, but often also keep livestock. Mobile pastoralists are livestock keeper, who sometimes cultivate food crops. Rabies is endemic in Chad with 20 to 90 persons dying from rabies every year according to the WHO [6]. Rabies incidence data in dog population are only available from the capital, N'Djaména[7,8].
		Rural 2	Sinetaye	Low		Region: Batha Department: Lake Fitri District: Yao	
Guatemala	Universidad del Valle de Guatemala (UVG)	Rural 1	La Romana	Low	Mai/June 2018	Department: Petén Municipality: Poptún	Petén department in Guatemala is scarcely populated with 69% Ladino (Spanish-speaking) and 30% Maya ethnicities[9]. The Petén Department's main economic activity involves large plantations of African palm and cattle farms, yet the majority of the population's economic activity refers to subsistence agriculture, with a very small sector involved in tourism activities[10]. The overall illiteracy rate is 22% and nearly half of the population is living under the poverty rate[9]. According to the WHO, two cases of human rabies were in Guatemala reported between 2013 and 2016, whereas no human rabies case was reported in Petén department since 1990[6]. From 2008 to 2014, five positive dogs were reported from the Petén Sur Oriental health area.
		Rural 2	Sabaneta	Medium			
		Urban	Poptún	High			

Indonesia	Kupang State Agricultural Polytechnics (KAP)	Rural 1	Pogon	Low	July 2018	Province: East Nusa Tenggara Island: Flores Regency: Sikka	The Sikka Regency in Indonesia is mainly populated by Catholics of the ethnicities Sikka, Krowe, Tana Ai, Palue and Lio (https://www.sikkakab.go.id/artikel-budaya). The main economic activity is agriculture[11]. Nearly half of people graduated from elementary school and live with less than US$52 per month[12]. According to the WHO, an estimated 150 to 300 human cases of rabies occur in Indonesia every year[13]. Flores Island, Indonesia, is endemic of canine rabies since 1998, and causes 15 human deaths annually[11,14]. During six month in 2019, 27 dogs were confirmed as rabies positive (https://mediaindonesia.com/nusantara/248367/wabah-rabies-menyebar-di-11-kecamatan-di-sikka).
		Rural 2	Hepang	Medium	August 2018		
		Semi-urban	Habi	High	July 2018		
Uganda	College of Veterinary Medicine, Animal Resources and Biosecurity (CoVAB) of Makerere University	Rural 1	Kamuda 1	Low	Jan/Feb 2019	Region: Eastern Uganda District: Soroti	Soroti district is located in Teso region in eastern part of Uganda, mainly inhabited the ethnic groups of Iteso and Kumam[15]. The main economic activity is subsistence mixed farming with the majority being small holders engaging 68.3% of the households. Other sources of income for households include small scale family businesses. Households in rural villages are scattered and grass thatched, while majority of houses in urban and peri-urban areas are built with walls, roof and floors mainly constructed with temporal materials[16]. Canine rabies is endemic in Uganda with an estimated 58 human dog bites per 100,000 persons[17] and 90 to 400 human deaths yearly[18]. Global alliance for rabies control reported a total of 62 dog rabies positive samples between 2015 and 2020 in Uganda.
		Rural 2	Kamuda 2	Low			
		Semi-urban	Soroti municipality	High			

Supplementary Table S39. List of the selected best models for the dog-level PBLM using log-transformed degree as output in Guatemala – urban. Blue: control variables, dark grey: variables selected in each model. Light grey: variable excluded from the model because data was not available (age) or all dogs had the same characteristics (none of the dogs were kept as hunting dogs nor raised for meat production).

Rank	Sex	Age	Low BCS	High BCS	Guardian	Shepherd	Hunting	Source of meat	FRT	NDC	Distance	delta AIC
1												0.00
2												0.35
3												1.50
4												1.78
5												1.86
6												1.88

Supplementary Questionnaire Form S1 (separate file). Questionnaire used for the owner’s interview in Chad

Supplementary Questionnaire Form S2 (separate file). Questionnaire used for the owner’s interview in Guatemala

Supplementary Questionnaire Form t S3 (separate file). Questionnaire used for the owner’s interview in Indonesia

Supplementary Questionnaire Form S4 (separate file). Questionnaire used for the owner’s interview in Uganda

Supplementary Dataset S1 (separate file). Information collected during the interviews in Chad

Supplementary Dataset S2 (separate file). Information collected during the interviews in Guatemala

Supplementary Dataset S3 (separate file). Information collected during the interviews in Indonesia

Supplementary Dataset S4 (separate file). Information collected during the interviews in Uganda

References

1. Schelling, E. *et al.* Health Research Among Highly Mobile Pastoralist Communities of Chad. *Soc. Biol. Hum. Aff.* **75**, 95–115 (2010).
2. Le, S., Josse, J. & Husson, F. FactoMineR: An R Package for Multivariate Analysis. *J. Stat. Softw.* **25**, 1–18 (2008).
3. R Core Team. R: A Language and Environment for Statistical Computing. (2020).
4. Warembourg, C. *et al.* Comparative study of free-roaming domestic dog management and roaming behavior across four countries: Chad, Guatemala, Indonesia and Uganda. *Front. Vet. Sci.* **8**, (2021).
5. Lechthaler, F. *et al.* Bottlenecks in the provision of antenatal care: rural settled and mobile pastoralist communities in Chad. *Trop. Med. Int. Heal.* **23**, 1033–1044 (2018).
6. World Health Organization. *WHO expert consultation on rabies*. *World Health Organization - Technical Report Series* (2018).
7. Dürr, S. *et al.* Rabies diagnosis for developing countries. *PLoS Negl. Trop. Dis.* **2**, (2008).
8. Zinsstag, J. *et al.* Vaccination of dogs in an African city interrupts rabies transmission and reduces human exposure. *Sci. Transl. Med.* **9**, (2017).
9. Warembourg, C. *et al.* Estimation of free-roaming domestic dog population size: Investigation of three methods including an Unmanned Aerial Vehicle (UAV) based approach. *PLoS One* **15**, 1–24 (2020).
10. MINECO. *Bandera Y Escudo Municipal Del Departamento De El Petén*. http://www.mineco.gob.gt/sites/default/files/peten_1.pdf (2017).
11. Wera, E., Mourits, M. C. M. & Hogeveen, H. Uptake of Rabies Control Measures by Dog Owners in Flores Island, Indonesia. *PLoS Negl. Trop. Dis.* **9**, 1–23 (2015).
12. Wera, E., Mourits, M. C. M. & Hogeveen, H. Intention of dog owners to participate in rabies control measures in Flores Island, Indonesia. *Prev. Vet. Med.* **126**, 138–150 (2016).
13. World Health Organisation (WHO). *Strategic Framework for Elimination of Human Rabies Transmitted by Dogs in the South-East Asia Region*. (2012).
14. Wera, E. Socio-economic modelling of rabies control in Flores Island, Indonesia. *PhD Thesis. Wageningen: Wageningen University* (2017).
15. Onapa, A. W., Simonsen, P. E., Pedersen, E. M. & Okello, D. O. Lymphatic filariasis in Uganda : baseline investigations districts. *Trans. R. Scoiety Trop. Hyg. Med.* 161–167 (2001).
16. Uganda Bureau of Statistics (UBOS). *The National Population and Housing Census 2014 - Main Report, Kampala, Uganda*. http://www.ubos.org (2016).
17. Masiira, B. *et al.* Long term trends and spatial distribution of animal bite injuries and deaths due to human rabies infection in Uganda, 2001-2015. *PLoS One* **13**, 2001–2015 (2018).
18. Hampson, K. *et al.* Estimating the Global Burden of Endemic Canine Rabies. *PLoS Negl. Trop. Dis.* **9**, 1–20 (2015).
